# Tetramethylpyrazine ameliorates endotoxin-induced acute lung injury by relieving Golgi stress via the Nrf2/HO-1 signaling pathway

**DOI:** 10.1186/s12890-023-02585-3

**Published:** 2023-08-07

**Authors:** Shaona Li, Yexiang Xu, Simeng He, Xiangyun Li, Jia Shi, Bing Zhang, Youzhuang Zhu, Xiangkun Li, Yanting Wang, Cuicui Liu, Yang Ma, Shuan Dong, Jianbo Yu

**Affiliations:** 1 Department of Anesthesiology and Critical Care Medicine, Tianjin Nankai Hospital, Tianjin Medical University, Tianjin, 300100 China; 2 Department of Anesthesiology, The Affiliated Hospital of Qingdao University, Qingdao, 266000 Shandong Province China; 3 Department of Anesthesiology, Qilu Hospital, Cheeloo College of Medicine, Shandong University, Jinan, 250000 Shandong Province China

**Keywords:** Tetramethylpyrazine (TMP), Endotoxin, Acute lung injury, Golgi stress, Nuclear factor erythroid-2 related factor 2 (Nrf2), Heme oxygenase-1 (HO-1)

## Abstract

**Purpose:**

Endotoxin-induced acute lung injury (ALI) is a severe disease caused by an imbalanced host response to infection. It is necessary to explore novel mechanisms for the treatment of endotoxin-induced ALI. In endotoxin-induced ALI, tetramethylpyrazine (TMP) provides protection through anti-inflammatory, anti-apoptosis, and anti-pyroptosis effects. However, the mechanism of action of TMP in endotoxin-induced ALI remains unclear. Here, we aimed to determine whether TMP can protect the lungs by inhibiting Golgi stress via the Nrf2/HO-1 pathway.

**Methods and results:**

Using lipopolysaccharide (LPS)-stimulated C57BL/6J mice and MLE12 alveolar epithelial cells, we observed that TMP pretreatment attenuated endotoxin-induced ALI. LPS + TMP group showed lesser lung pathological damage and a lower rate of apoptotic lung cells than LPS group. Moreover, LPS + TMP group also showed decreased levels of inflammatory factors and oxidative stress damage than LPS group (*P* < 0.05). Additionally, LPS + TMP group presented reduced Golgi stress by increasing the Golgi matrix protein 130 (GM130), Golgi apparatus Ca^2+^/Mn^2+^ ATPases (ATP2C1), and Golgin97 expression while decreasing the Golgi phosphoprotein 3 (GOLPH3) expression than LPS group (*P* < 0.05). Furthermore, TMP pretreatment promoted Nrf2 and HO-1 expression (*P* < 0.05). Nrf2-knockout mice or Nrf2 siRNA-transfected MLE12 cells were pretreated with TMP to explore how the Nrf2/HO-1 pathway affected TMP-mediated Golgi stress in endotoxin-induced ALI models. We observed that *Nrf2* gene silencing partially reversed the alleviating effect of Golgi stress and the pulmonary protective effect of TMP.

**Conclusion:**

Our findings showed that TMP therapy reduced endotoxin-induced ALI by suppressing Golgi stress via the Nrf2/HO-1 signaling pathway in vivo and in vitro.

**Supplementary Information:**

The online version contains supplementary material available at 10.1186/s12890-023-02585-3.

## Introduction

Sepsis is a syndrome of dysregulated systemic inflammatory response triggered by infection [1]. Patients with sepsis are extremely sensitive to acute lung injury (ALI) and acute respiratory distress syndrome (ARDS) [2, 3]. Sepsis-induced ALI is characterized primarily by an uncontrolled cascade of inflammatory reactions [4–6]. Clinical strategies for treating sepsis-induced ALI mainly include symptomatic supportive methods, but specific drugs or therapeutic strategies are still lacking [7]. Therefore, it is necessary to explore novel mechanisms for the treatment of sepsis-induced ALI.

Essential for intracellular lipid and protein transport, the Golgi apparatus (GA) has received increasing attention in inflammatory diseases in recent years [8, 9]. The concept of Golgi stress was first introduced in *Free Radical Biology and Medicine* in a study reporting the adaptive response of GA to harmful stimuli [10]. Researchers discovered that Golgi stress is linked to several disorders, including inflammatory diseases and cancer [11, 12]. Moreover, our previous research revealed that Golgi stress is implicated in sepsis-induced ALI and may be modulated by heme oxygenase-1 (HO-1) [13]. Therefore, modulation of Golgi stress may provide a novel approach for healing sepsis-induced ALI.

The Nrf2/HO-1 pathway provides anti-inflammatory and antioxidative benefits in oxidative stress diseases [14]. Nuclear factor red lineage-2 related factor-2 (Nrf2) activity is typically suppressed by specific binding to the cytosolic keap1 chaperone protein [15]. When stimulated by oxidative stress products, Nrf2 disintegrates from keap1, transfers from the cytoplasm to the nucleus, and increases the ARE response [16, 17]. As a significant protein induced by the Nrf2-ARE response, HO-1 facilitates heme degradation into biliverdin and CO. These endogenous protective chemicals control various cellular functions, including oxidation and apoptosis [18].

Tetramethylpyrazine (TMP) is a bioactive alkaloid originating from the Chinese plant chuanxiong. TMP is frequently utilized in clinical practice to treat vascular disorders because of its multiple functions, such as antiplatelet effects [19, 20]. Notably, TMP is progressively gaining attention for its anti-inflammatory and antioxidative benefits [21, 22]. Jiang et al. [23] found that TMP could ameliorate LPS-induced ALI by suppressing apoptosis and pyroptosis. TMP has previously been shown to ameliorate motor dysfunction by stimulating the PGC-1/Nrf2/HO-1 pathway [24]. Moreover, researchers have found that activating HO-1 reduces endotoxin-induced ALI by mitigating Golgi stress [13, 25].

Using LPS-stimulated ALI animals and the MLE12 cell model, we aimed to investigate whether TMP pretreatment prevents endotoxin-induced ALI by reducing Golgi stress. Then, we applied Nrf2 knockout or knockdown technologies to determine whether the Nrf2/HO-1 signaling pathway is related to the capacity of TMP to decrease Golgi stress and ameliorate endotoxin-induced ALI.

## Methods

### Animal preparation

Beijing Huafukang Biotechnology Co. supplied 6–8-week-old male C57BL/6J mice weighing 20–25 g. Jiangsu Huachuang Sino Pharmaceutical Technology Co. supplied well-characterized Nrf2-knockout (Nrf2 KO) male C57BL/6J mice (6–8-week-old, weighing 20–25 g). All animal experiments were conducted in accordance with Tianjin Medical Experimental Animal Care standards, and animal operations were approved by the Animal Care and Use Committee of Tianjin Nankai Hospital (Approval NO. NKYY-DWLL-2022–049). The mice were housed in their own cages in a clean and well-ventilated animal environment with SPF conditions of 22–24 °C and 60–65% humidity. Mice were given unrestricted access to food and water and kept on a 12-h day/night cycle. All animals were anesthetized with 35 mg/kg intraperitoneal injection of 0.2% pentobarbital sodium, and anesthesia was sustained by 1–3% isoflurane breathing. The absence of the skin pinch response or toe squeeze reflex and relaxation of the mice's head, neck, and limb muscles indicated the correct level of anesthesia.

To study the effect of TMP on LPS-induced ALI, mice were randomly assigned to four groups (*n* = 6/group): control group, LPS group, LPS + TMP group, and TMP group. In addition, to study the effect of Nrf2/HO-1 pathway in the TMP-mediated endotoxin-induced ALI, WT and Nrf2 KO mice were assigned to six groups (*n* = 6/group): WT + control group, Nrf2 KO + control group, WT + LPS group, Nrf2 KO + LPS group, WT + LPS + TMP group, and Nrf2 KO + LPS + TMP group. According to a previous study, 15 mg/kg LPS (*Escherichia coli*, O55:B5 Sigma USA) [26] diluted in 0.2 ml saline was administered through the caudal vein to mice to create models of endotoxin-induced ALI in vivo. Mice in the LPS + TMP group (both WT and Nrf2 KO mice) were administered 50 mg/kg TMP (Harbin Sanlian Pharmaceutical Co., Ltd. Harbin, China) [27] intraperitoneally 1 h before LPS administration. Mice in the TMP group were intraperitoneally administered 50 mg/kg TMP without LPS stimulus. Equal amounts of saline were administered to the control groups of WT and Nrf2 KO mice. The mice were sacrificed by bloodletting and sodium pentobarbital overdose after 12 h of careful observation. Serum was collected from blood samples centrifuged at 3000 g for 30 min at 4°C following immediate heart puncture. Additionally, the lung tissues were placed in liquid nitrogen or − 80°C freezing for further biochemical analysis or histological research. Table 1 provides an overview of mouse handling and categorization.

**Table 1:** Animal experimental treatments and groupings

Grouping	Treatment
Effects of TMP on LPS-induced ALI in C57BL/6 J mice (*n* = 6/group)	
Control	Sham operation plus normal saline
LPS	Caudal vein injection of 15 mg/kg LPS diluted in 0.2 ml saline for 12 h
LPS + TMP	50 mg/kg TMP was pretreated intraperitoneally (i.p.) 1 h prior to LPS challenge
TMP	Sham operation plus 50 mg/kg TMP
Effects of Nrf2/HO-1 pathway on Golgi stress during TMP attenuates lung injury in Nrf2 knockout (Nrf2 KO) and WT mice (*n* = 6/group)	
WT + Control	WT mice + Sham operation plus normal saline
Nrf2 KO + Control	Nrf2 KO mice + Sham operation plus normal saline
WT + LPS	WT mice + Caudal vein injection of 15 mg/kg LPS diluted in 0.2 ml saline for 12 h
Nrf2 KO + LPS	Nrf2 KO mice + Caudal vein injection of 15 mg/kg LPS diluted in 0.2 ml saline for 12 h
WT + LPS + TMP	WT mice + 50 mg/kg TMP were intraperitoneally (i.p.) administered 1 h prior to LPS challenge
Nrf2 KO + LPS + TMP	Nrf2 KO mice + 50 mg/kg TMP was intraperitoneally (i.p.) administered 1 h prior to LPS challenge

*Abbreviations*: *TMP* Tetramethylpyrazine, *LPS* lipopolysaccharide, *ALI* acute lung injury, *Nrf2* nuclear factor erythroid 2-related factor-2, *HO-1* heme oxygenase-1

### Histological analysis

The acquired lung tissues were kept in a 10% paraformaldehyde solution for 24 h. After routine dehydration, cleaning, and embedding in paraffin, the tissues were cut into 4-mm slices and stained with hematoxylin and eosin (H&E). Using the BX51-P light microscope (Olympus, Japan), lung tissue slices were examined by H&E staining. A semiquantitative scoring system based on the following parameters was used to assess the level of lung injury: misconfiguration of the lung parenchyma, pulmonary edema, neutrophil infiltration, and hemorrhage. In brief, the following rating scale was employed: 0 = no or little harm, 1 = light damage (25%), 2 = moderate damage (50%), 3 = severe damage (75%), and 4 = extremely severe damage (approximately 100%). As previously indicated, the degree of lesions was assessed for all samples, and the lung injury scores of the sections were calculated by adding the values of each criterion. Two pathologists who were not acquainted with the experimental setting assessed the lung injury scores.

### Wet-to-dry weight (W/D) ratio

We collected the right lung tissues and cleaned their surface water using filter paper. We used an electronic scale to calculate the weight of the tissues and calculated the wet weight. Then, we dried the right lung tissues in an oven at 70°C for 48–72 h until the weight remained constant, and the measured value served as the dry weight. The formula for calculating W/D was W/D = wet weight/dry weight.

### TUNEL assay

Staining was performed using the DeadEndTM Fluorescent TUNEL System Kit (Roche, USA). Slices were deparaffinized and treated for 8–10 min with protease K (20 g/ml in PBS). TUNEL labeling buffer (a mixture of 5 μL nucleotide mix, 45 μL of equilibration buffer, and 1 μL rTdT enzyme) was administered, and the samples were incubated for 1 h at 37°C in a humidified lucifugal box. The slides were protected from light until the experiment was complete. Hematoxylin was used to stain the cell nuclei for 10 min. Images were acquired using Image-Proplus (Media Cybernetics, USA) after mounting the slides with an antifade solution.

### Enzyme-linked immunosorbent assay (ELISA) quantification

Using ELISA kits (SEKM-0002, SEKM-0007, and SEKM-0034; Beijing Solarbio Science & Technology Co., Ltd., China), we measured the levels of IL-1, IL-6, and TNF- in the serum or culture supernatants. Testing was performed according to the manufacturer’s instructions.

### Measurement of oxidative stress

An appropriate quantity of lung tissue was collected to prepare lung tissue homogenate, and the supernatant was obtained for testing. GSH and GSSG levels were measured using a T-GSH/GSSG assay kit (A061-1; Nanjing Jiancheng Institute of Biological Engineering, China). GSH and GSSG units are reported in μmol/L. GSH/GSSG denotes the GSH to GSSG ratio. Malondialdehyde (MDA) content in the cell supernatant was determined using an MDA assay kit (A003-2–2; Wuhan Swinbio Biotechnology Co., China) and reported as nmol/ml. Superoxide dismutase (SOD) activity in the cell supernatant was determined using a SOD assay kit (A001-3, Nanjing Jiancheng Institute of Biological Engineering, China) and reported as U/ml.

### Immunofluorescence staining

Lung tissue slices were fixed in 4% paraformaldehyde for 10 min before rinsing twice with PBS. These slices were incubated overnight with primary antibody and then for an additional hour with fluorescein-coupled secondary antibody. Anti-GM130 antibody (Boster, M05865-2) and 6-diamino-2-phenylindole (DAPI) were used to identify GA and nuclear structures. A Nikon confocal fluorescence microscope was used to obtain fluorescence images.

### Western blotting

Lung tissues or cells were homogenized using a lysis buffer on ice. The cell lysates were centrifuged at 12000 rpm for 10 min at 4°C before the supernatant was collected. The protein concentrations were detected with the BCA method (Thermo, USA). 50 μg/per well protein extracts were used for electrophoresis by 10% SDS-PAGE and transferred to PVDF membranes (Bio-Rad, USA). Membranes were horizontally cut to probe proteins with different molecular weights. The membranes were not stripped or reprobed. After blocking in Tris-buffered saline with 5% nonfat powdered milk for 1 h at 37℃, the membranes were incubated with primary antibodies against Nrf2 (1:500, Cell Signaling, #12721), HO-1 (1:10000, Abcam, ab68477), GM130 (1:800, Boster, M05865-2), Golgin 97(1:1000, Absin, abs122617), ATP2C1 (1:500, Proteintech, 13310–1-AP), GOLPH3 (1:1000, Abcam, ab98023), and β-actin (1:5000, ZSGB-BIO, TA-09) at 4°C overnight. After three washes with TBST (10 min each), the blots were incubated with the appropriate secondary antibody for 1 h. Given its constitutive expression, β-actin was used as the loading control. The blots were visualized with an enhanced chemiluminescence system (Bio-Rad) and quantified using ImageJ (V1.8.0.112).

### Cell treatment

The mouse lung epithelial cell line MLE12 was grown in the HITES medium in a 5% CO_2_ atmosphere at 37°C for 24 h before being seeded into 96-well plates at 5 × 10^4^ cells/well. Incubation was extended for 24 h in the presence of various doses of LPS (0, 0.1, 0.5, 1, 5, and 10 μg/ml). Different doses of TMP (0, 2.5, 5, 10, 50, and 100 μg/ml) were added 1 h before LPS administration and incubated for 24 h. The Cell Counting Kit-8 (CCK-8) assay was performed to select the most effective TMP concentration for LPS-stimulated cells. For additional experiments, MLE12 cells were cultivated in six-well plates during logarithmic growth. Next, these cells were randomized and split into five groups: Cells in the LPS group were incubated with LPS (5 μg/ml) for 24 h. Cells in the LPS + TMP group were pre-incubated with TMP (50 μg/ml) for 1 h prior to LPS (5 μg/ml) treatment for 24 h. The control group was administered an equivalent volume of saline solution. For 48 h, the Nrf2 siRNA + LPS + TMP and NC siRNA + LPS + TMP groups were transfected with Nrf2 and NC siRNA, respectively. Then, 50 μg/ml LPS was added, and 5 μg/ml LPS was added after 1 h. The incubation was continued for 24 h. Supernatants from the cultivated cells were then obtained for biochemical analysis, and the cells were collected for western blotting. The grouping and treatment of the cells are shown in Table 2.

**Table 2:** Cell experimental treatments and groupings

Grouping	Treatment
Effects of the Nrf2/HO-1 pathway on TMP-mediated Golgi stress during LPS-induced oxidative injury in Nrf2 siRNA and NC siRNA-transfected MLE12 cells (*n* = 6 per group)	
Control	Cells are cultured normally in medium
LPS	5 μg/ml LPS incubated for 24 h
LPS + TMP	50 μg/ml TMP pre-incubated 1 h prior to 5 μg/ml LPS for 24 h
Nrf2 siRNA + LPS + TMP	50 μg/ml TMP pre-incubated 1 h prior to 5 μg/ml LPS for 24 h in Nrf2 siRNA-transfected MLE12 cells
NC siRNA + LPS + TMP	50 μg/ml TMP pre-incubated 1 h prior to 5 μg/ml LPS for 24 h in NC siRNA-transfected MLE12 cells

*Abbreviations*: *TMP* Tetramethylpyrazine, *LPS* lipopolysaccharide, *ALI* acute lung injury, *Nrf2* nuclear factor erythroid 2-related factor-2, *HO-1* heme oxygenase-1

### Cell viability

To determine the viability of MLE12 cells exposed to LPS with or without TMP, we used the CCK-8 assay (Beyotime, Shanghai, China). In short, 10 μl CCK-8 solution was added to each well and then incubated at 37°C for 2.5 h. After mixing the wells, absorbance was recorded at 450 nm using a microplate reader (Bio-Rad, Hercules, CA, USA).

### Transient transfection with siRNA

Nrf2 and NC siRNA were designed and synthesized by Suzhou GenePharma Co. Ltd. For transfection experiments, 5 × 10^5^/ml MLE12 cells were inoculated in six-well plates. Cells were transfected with siRNA mixed with siRNA-mate reagent in DMEM high-sugar medium (Wolcavi Biotechnology Co. Beijing. China). Cells were incubated at 37 °C and collected after 48 h to detect the level of gene silencing using RT-PCR.

### Statistical analysis

All values are presented as mean ± standard error of the mean (SEM). A paired *t-*test was used to compare significant differences between the two groups. To compare multiple groups, GraphPad Prism 9.2.0 (GraphPad Software, La Jolla, CA) was used for one-way analysis of variance followed by the Bonferroni post-test. Statistical significance was defined as *P*-values < 0.05 (*P < 0.05; **P < 0.01; ***P < 0.001).

## Results

### TMP ameliorated LPS-driven lung injury in vivo

**Fig. 1:**
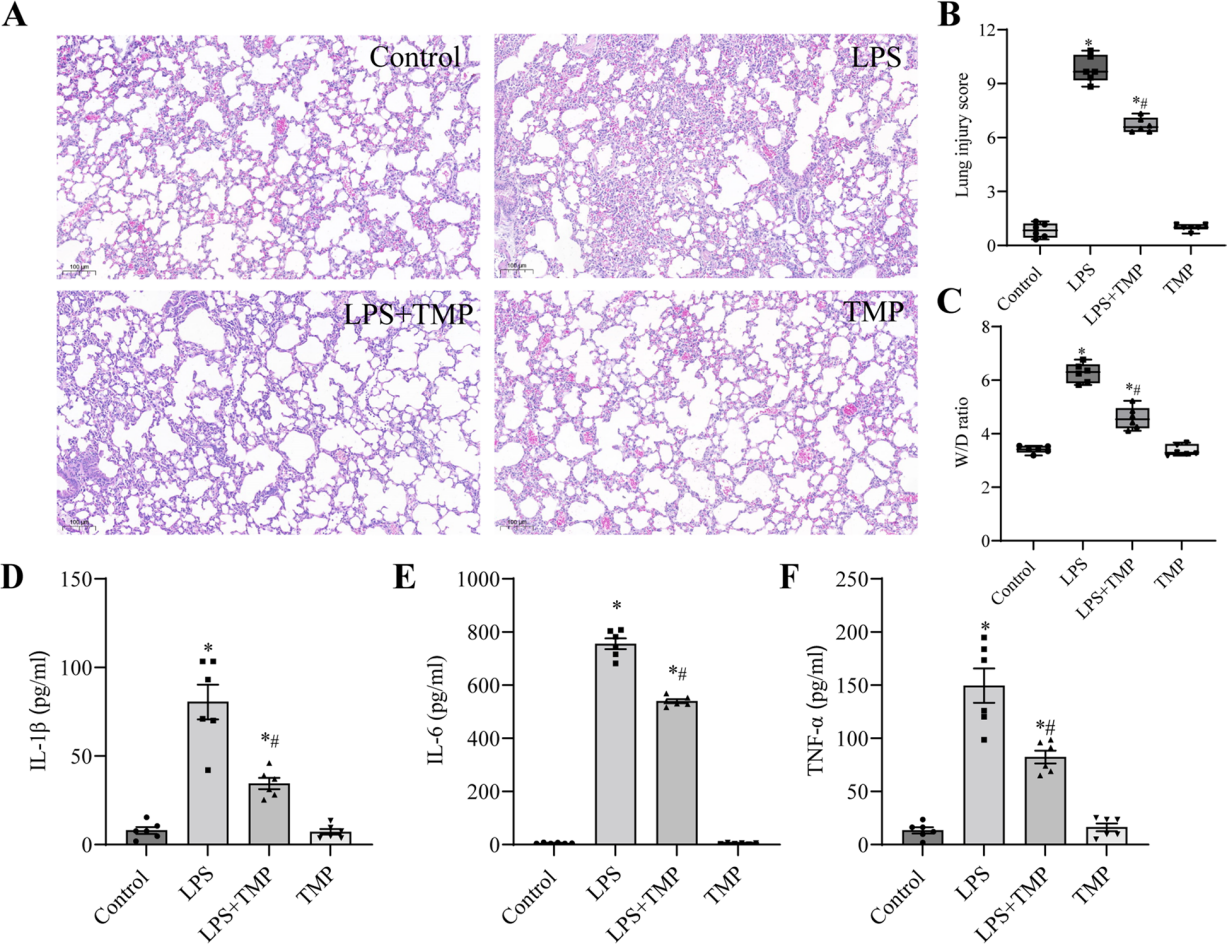
TMP pretreatment attenuated LPS-induced ALI in mice. **A** HE staining was applied to assess the histopathological changes in the lung sections of LPS-stimulated ALI mice pretreated with or without TMP (original magnification, × 200). Scale bar: 100 μm. **B** The lung injury scores were evaluated by two blinded pathologists to determine the degree of lung injury. **C** The lung wet/dry (W/D) weight ratio. **D**-**F** Proinflammatory cytokine IL-1β, IL-6, and TNF-α levels in the serum were detected using ELISA. Data for the bar graphs are presented as mean ± SEM, and multiple comparisons were performed using one-way ANOVA with Bonferroni coefficient (*n* = 6). *Significant difference compared with the control group, *P < 0.05; ^#^Significant difference compared with the LPS group, *P* < 0.05. ALI: acute lung injury; ANOVA: analysis of variance; ELISA: enzyme-linked immunosorbent assay; HE: hematoxylin and eosin; LPS: lipopolysaccharide; SEM, standard error of the mean; TMP: tetramethylpyrazine

We investigated the pathological changes in lung specimens, lung injury scores, and the W/D ratio in each group to confirm the impact of TMP on LPS-induced ALI. Preliminary observation of lung specimens in LPS group revealed noticeable pathological alterations, thickening of alveolar walls, pulmonary edema, severe infiltration of leukocytes, and hemorrhage, which were all significantly ameliorated in TMP-pretreated mice (Fig. 1A). Two blinded pathologists assessed the lung injury scores. Using the semiquantitative system of lung injury scoring, we obtained unanimous results. The lung injury score was considerably higher in LPS group than in control group; however, TMP pretreatment dramatically decreased this score (Fig. 1B). Noncardiogenic pulmonary edema was assessed using the lung W/D ratio. The W/D ratio was significantly higher in LPS group than in control group, and the W/D ratio was significantly lower in LPS + TMP group than in LPS group (Fig. 1C).

To investigate the substantial therapeutic impact of TMP on endotoxin-induced ALI, we measured the serum levels of proinflammatory cytokines IL-1β, IL-6, and TNF-1α. As shown in Fig. 1D-1F, exposure to LPS caused an apparent increase in IL-1β, IL-6, and TNF-α levels in mice, and TMP administration dramatically reduced these inflammatory factors. Therefore, we concluded that TMP ameliorated LPS-induced pulmonary pathological damage and inflammatory responses in mice. No significant difference was noted between TMP and control groups.

### TMP ameliorated apoptosis and oxidative stress in mice with LPS-induced ALI

**Fig. 2:**
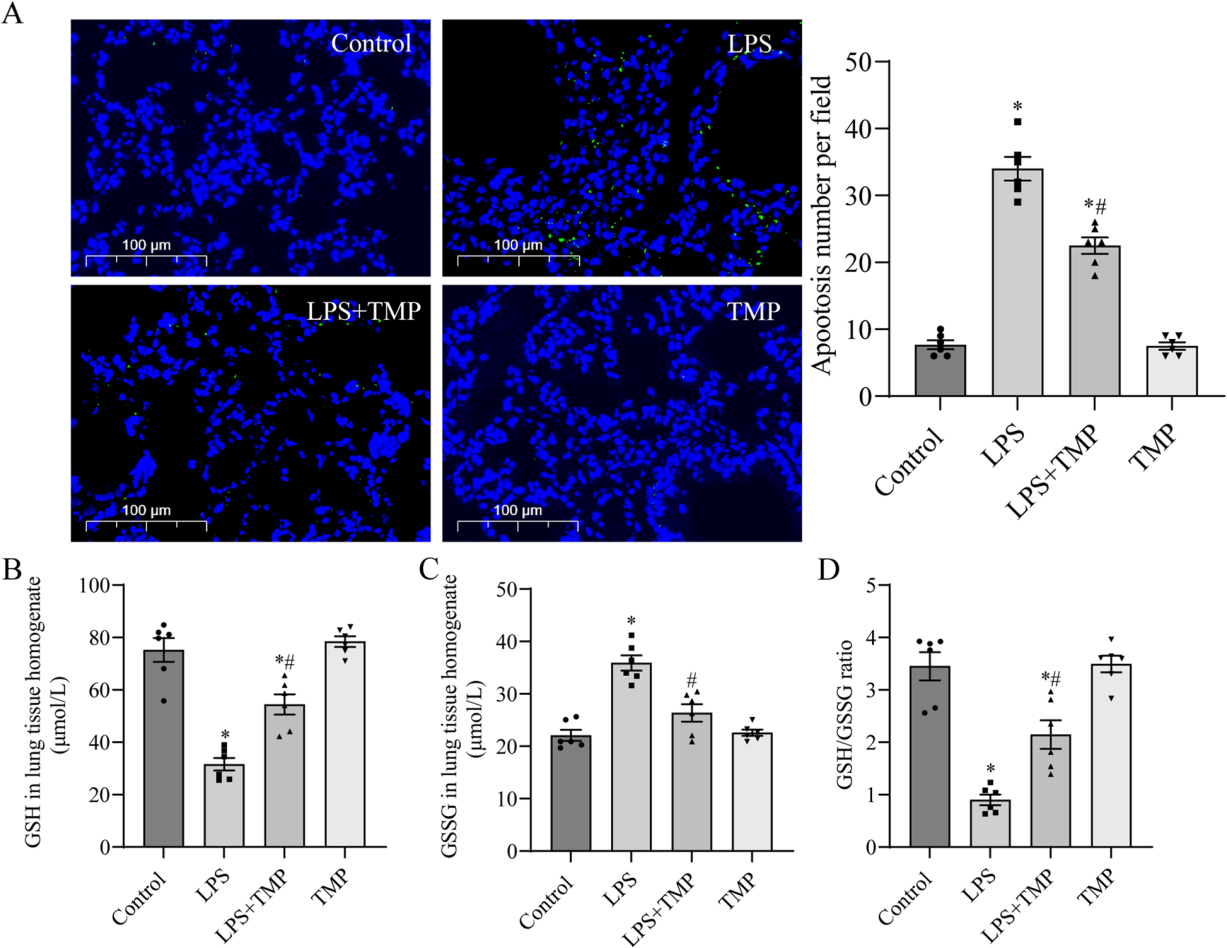
TMP pretreatment alleviated apoptosis and oxidative stress in LPS-stimulated mice. **A** TUNEL staining for apoptosis in lung tissues. The number of TUNEL-positive cells was determined by a blinded pathologist (original magnification, × 400). Scale bar: 100 μm. **B**, **C** GSH and GSSG levels were detected using T-GSH/GSSG assay kit. **D** GSH/GSSG ratio. Data for the bar graphs are presented as mean ± SEM, and multiple comparisons were performed using one-way ANOVA with Bonferroni coefficient (*n* = 6). *Significant difference compared with the control group, *P < 0.05; ^#^Significant difference compared with the LPS group, *P* < 0.05. ANOVA: analysis of variance; GSH: glutathione; GSSG: oxidized glutathione; LPS: lipopolysaccharide; SEM: standard error of the mean; TMP: tetramethylpyrazine

We used TUNEL staining to assess apoptosis in lung tissues (Fig. 2A). The quantity of TUNEL-positive cells was substantially lower in control and LPS + TMP groups than in LPS group. Thus, LPS stimulation exacerbated apoptosis in the lung tissue, which could be mitigated by TMP pretreatment.

The serum levels of GSH and GSSG and the GSH/GSSG ratio are the most common indicators of the response to oxidative damage. GSH levels and the GSH/GSSG ratio in lung tissues were lower, whereas GSSG levels were higher in LPS group than in control group (Fig. 2B-D). Compared with LPS group, TMP preconditioning markedly increased GSH levels and the GSH/GSSG ratio and decreased GSSG levels in the lung tissue. Thus, TMP decreased apoptosis and oxidative stress in mice exposed to LPS. In addition, the experimental results of TMP group did not differ substantially from those of control group.

### TMP alleviated Golgi stress and activated the Nrf2/HO-1 signaling pathway following LPS stimulation in vivo

**Fig. 3:**
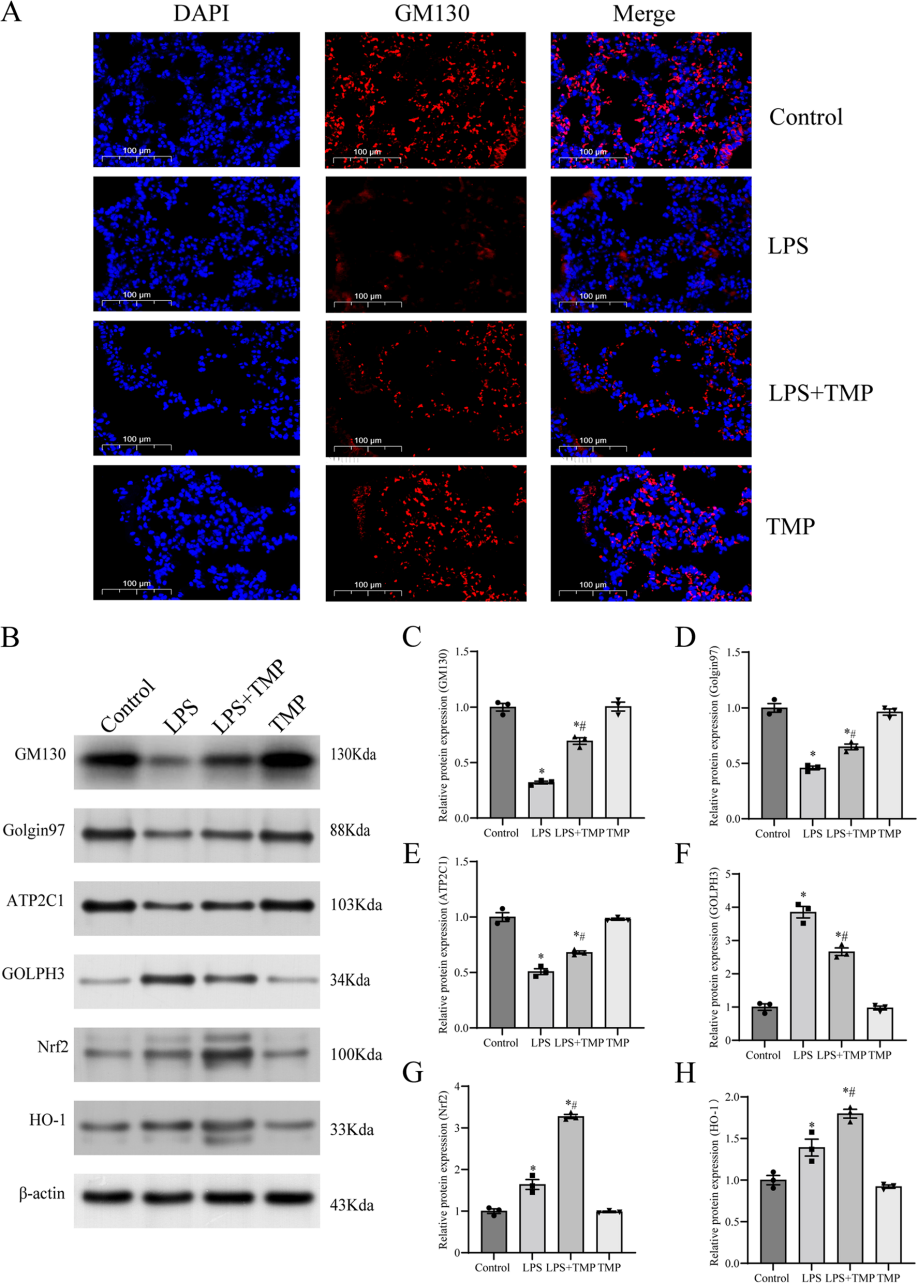
TMP pretreatment mitigated Golgi stress and activated the Nrf2/HO-1 pathway in the LPS-stimulated lung tissues of mice. **A** Immunofluorescence assays of GM130 protein by fluorescence microscope (original magnification, × 400). Scale bar: 100 μm. Red stood was used for FITC-GM130 stained sections, and blue stood was used for DAPI-stained nuclear structure. **B**-**H** Bands and semiquantification of western blotting to evaluate the expression of Golgi stress-related (GM130, Golgin97, ATP2C1, and GOLPH3) and pathway-related (Nrf2 and HO-1) proteins in the lung tissues of mice (*n* = 3 mice/group). The blots were cropped for improving the clarity and conciseness of the presentation. Full-length blots were presented in Additional file 1. Band intensity analysis on western blotting images shows their relative ratio to β-actin. Values from three independent samples are expressed as mean ± SEM, and multiple comparisons were performed using one-way ANOVA with the Bonferroni coefficient. *Significant difference compared with the control group, *P < 0.05; ^#^Significant difference compared with the LPS group, *P* < 0.05. ANOVA: analysis of variance; DAPI: 6-diamino-2-phenylindole; HO-1: heme oxygenase-1; LPS: lipopolysaccharide; Nrf2: nuclear factor-erythroid 2-related factor 2; SEM: standard error of the mean; TMP: tetramethylpyrazine

Double immunofluorescence (IF) using GM130 antibody (FITC-labeling, red) and DAPI (nuclear staining, blue) was used to explore morphological changes in GA. Compared with control group, the red fluorescence in LPS group became weaker and more diffuse, whereas TMP pretreatment alleviated this change (Fig. 3A). Then, we evaluated GM130, Golgin97, ATP2C1, and GOLPH3 protein expression using western blotting to evaluate the level of Golgi stress. Compared with control group, LPS treatment downregulated GM130, Golgin97, and ATP2C1 protein expression and upregulated GOLPH3 protein expression (Fig. 3B-F). Nevertheless, TMP pretreatment partially attenuated this effect. The results showed that TMP pretreatment ameliorated LPS-induced Golgi stress exacerbated by LPS. TMP without LPS stimulation had no impact on these variables compared with control group.

To further explore whether TMP ameliorates endotoxin-induced ALI and Golgi stress via the Nrf2/HO-1 pathway, we assessed Nrf2 and HO-1 protein expression. TMP pretreatment significantly upregulated Nrf2 and HO-1 expression compared with those in LPS group (Fig. 3B, G, H). Thus, we surmised that the preventive benefits of TMP against endotoxin-induced ALI may be mediated via the Nrf2/HO-1 pathway.

### Nrf2 KO partially offsetted the protective effects of TMP induced by LPS on mice

Nrf2 KO mice were treated with or without LPS or TMP using the same method as that used for wild-type mice. Nrf2 KO + LPS mice showed more severe lung histopathological injury, increased inflammatory response, and greater oxidative damage than WT + LPS mice. These results validated the role of Nrf2 as a protective regulator against endotoxin-induced ALI.

**Fig. 4:**
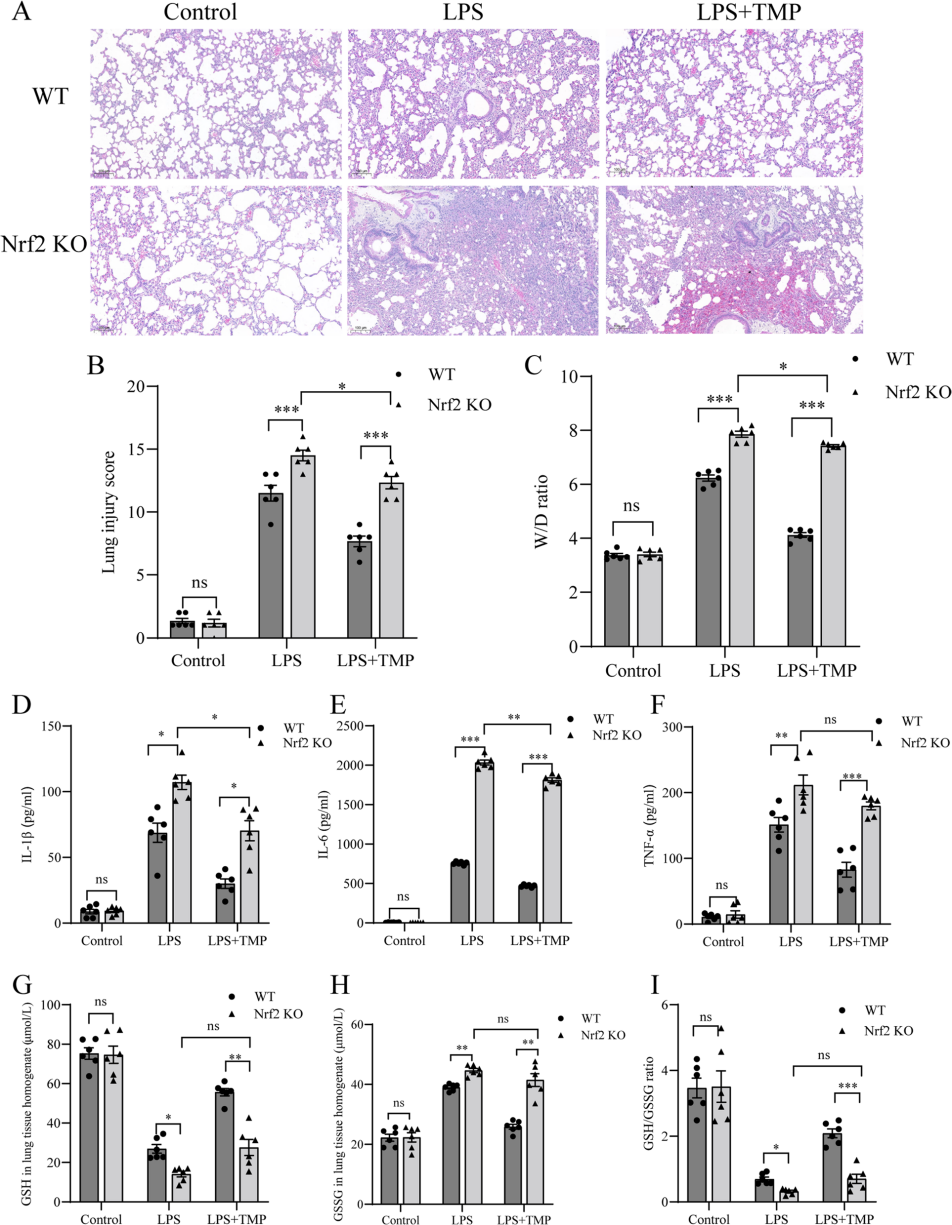
Nrf2 KO partially counteracted the TMP-mediated lung protective effects in LPS-induced lung injury in mice. **A** The representative histopathological changes (HE staining) of the lung (original magnification × 200). Scale bar: 100 μm. **B** The lung injury scores were evaluated by two blinded pathologists to determine the degree of lung injury. **C** The lung wet/dry (W/D) weight ratio. **D**-**F** Proinflammatory cytokines IL-1β, IL-6, and TNF-α levels in the serum were detected using ELISA. **G**-**H** GSH and GSSG levels were detected using T-GSH/GSSG assay kit. **I** The GSH/GSSG ratio. Data for the bar graphs are presented as mean ± SEM, and multiple comparisons were performed using one-way ANOVA with Bonferroni coefficient (*n* =  6/group). ns = no statistical difference, ^*^ *P* < 0.05, ^**^ *P* < 0.01, ^***^ *P* < 0.001. ANOVA: analysis of variance; ELISA: enzyme-linked immunosorbent assay; GSH: glutathione; GSSG: oxidized glutathione; HE: hematoxylin and eosin; KO: knockout; LPS: lipopolysaccharide; Nrf2: nuclear factor-erythroid 2-related factor 2; TMP: tetramethylpyrazine; WT: wild-type

To further elucidate whether TMP acts as a lung-protective factor via the Nrf2/HO-1 pathway, we compared Nrf2 KO + LPS + TMP group with WT + LPS + TMP group. Thickened alveolar walls, alveolar hemorrhage, and neutrophil infiltration were more severe in Nrf2 KO + LPS + TMP group than in WT + LPS + TMP group (Fig. 4A). The lung injury scores and W/D ratio were significantly higher in Nrf2 KO + LPS + TMP group than in WT + LPS + TMP group (Fig. 4B, C). Moreover, IL-1β, IL-6, and TNF-α levels were increased by 2.45, 3.86, and 2.17 times, respectively, in Nrf2 KO + LPS + TMP group compared with WT + LPS + MP group (Fig. 4D–F). The oxidative stress indicators GSH level and the GSH/GSSG ratio were significantly lower in Nrf2 KO + LPS + TMP group than in WT + LPS + TMP group (Fig. 4G-I).

We concluded that Nrf2 KO partially counteracted the protective effect of TMP towards endotoxin-induced ALI. Specifically, TMP attenuated endotoxin-induced ALI via the Nrf2 pathway. Moreover, in LPS-stimulated Nrf2 KO mice, pretreatment with TMP still partially attenuated lung injury and inflammation.

### Nrf2 KO mice were used to identify TMP-mediated lung protection by alleviating Golgi stress via the Nrf2/HO-1 signaling pathway

**Fig. 5:**
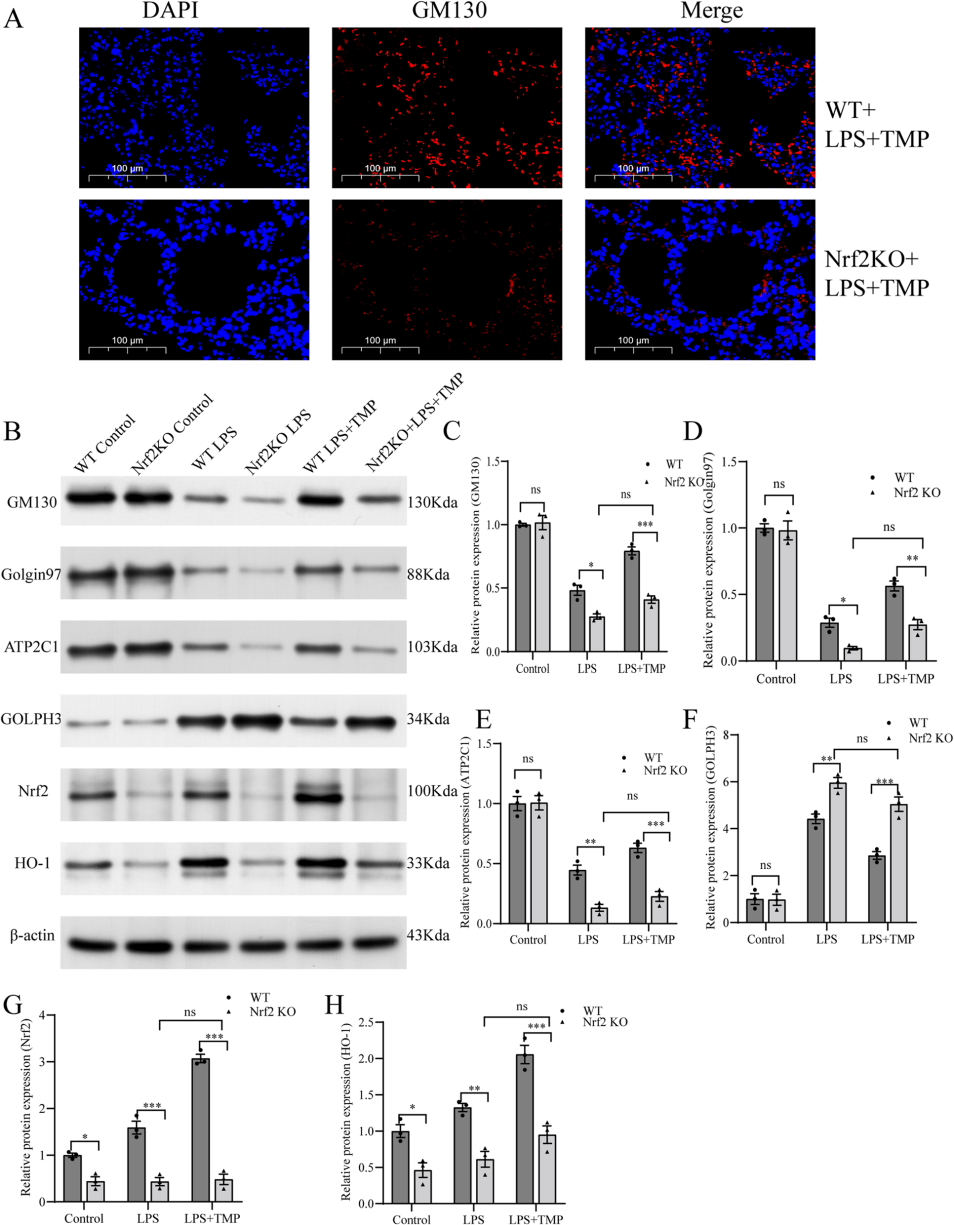
Blocking of the Nrf2/HO-1 pathway by Nrf2 KO partially counteracted the effects of TMP-afforded preservation of Golgi stress in endotoxin-induced ALI. **A** Immunofluorescence staining to observe GM130 protein expression in lung tissue (original magnification, × 400). Scale bar: 100 μm. **B**-**H** Bands and semi-quantification of western blotting to evaluate the expression of Golgi stress-related (GM130, Golgin97, ATP2C1, and GOLPH3) and pathway-related (Nrf2 and HO-1) proteins of lung tissues in mice. The blots were cropped for improving the clarity and conciseness of the presentation. Full-length blots were presented in Additional file 2. Band intensity analysis on western blotting images shows their relative ratio to β-actin. Values from three independent samples are presented as mean ± SEM, and multiple comparisons were performed using one-way ANOVA with the Bonferroni coefficient. ns = no statistical difference, ^*^ *P* < 0.05, ^**^ *P* < 0.01, ^***^ *P* < 0.001. ANOVA: analysis of variance; HO-1: heme oxygenase-1; KO: knockout; Nrf2: nuclear factor-erythroid 2-related factor 2; SEM: standard error of the mean; TMP: tetramethylpyrazine; WT: wild-type

To investigate the effect of the Nrf2 pathway on TMP pretreatment to attenuate Golgi stress, we used double IF with GM130 antibody and DAPI. Compared with WT + LPS + TMP mice, we observed decreased fluorescence intensity of FITC-GM130 in Nrf2 KO + LPS + TMP group (Fig. 5A).

The protein content of Golgi stress-related proteins GM130, Golgin97, ATP2C1, and GOLPH3 was evaluated using western blotting. Compared with WT + LPS group, Nrf2 KO + LPS group had decreased GM130, Golgin97, and ATP2C1 levels and increased GOLPH3 levels, suggesting that Nrf2 KO aggravated LPS-induced Golgi stress (Fig. 5B-F). Compared with WT + LPS + TMP mice, Nrf2KO + LPS + TMP mice showed a more remarkable decrease in the protein content of GM130, Golgin97, and ATP2C1 and increased content of GOLPH3. Specifically, the alleviation of Golgi stress by TMP in LPS-stimulated ALI was partially offset by Nrf2 knockdown. Nrf2 and HO-1 protein expression was significantly downregulated in the Nrf2 KO groups compared with the WT groups (Fig. 5B, G, H).

We concluded that TMP acted partially via the Nrf2/HO-1 pathway to ameliorate Golgi stress, thereby alleviating endotoxin-induced ALI. Notably, Nrf2-deficient mice pretreated with LPS or LPS + TMP exhibited similar levels of attenuation of Golgi stress.

### TMP attenuated inflammation and oxidative stress partially via the Nrf2/HO-1 pathway in LPS-stimulated MLE12 cells

**Fig. 6:**
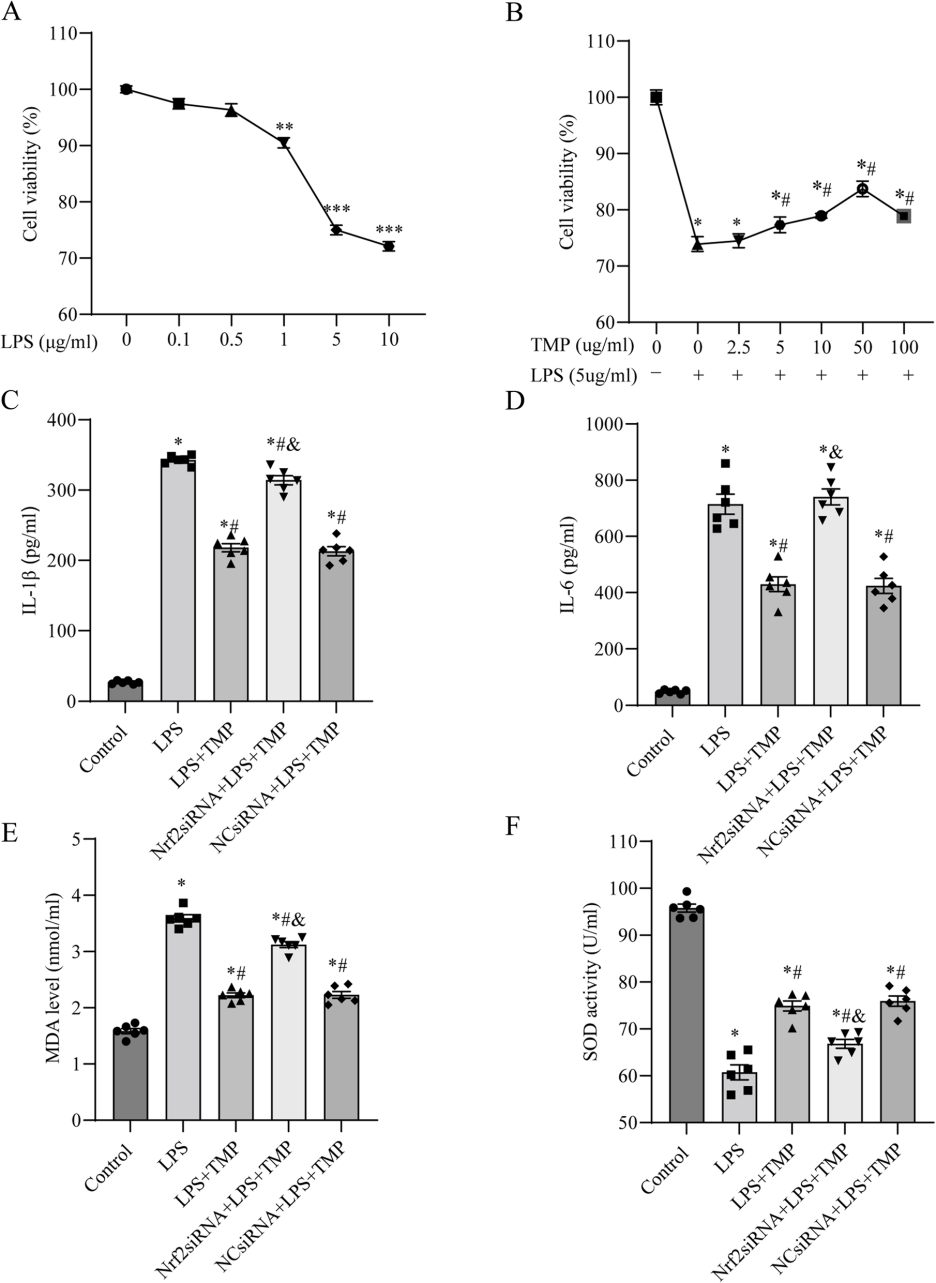
Nrf2 knockdown counteracted the effects of TMP-mitigated LPS-stimulated inflammation and oxidative damage in MLE12 cells. MLE12 cells were incubated with different LPS concentrations for 24 h, followed by a CCK-8 assay to determine cell viability. Data from five individual experiments were analyzed using one-way ANOVA and Bonferroni correction. Significant differences from the LPS = 0 group: ^*^ *P* < 0.05, ^**^ *P* < 0.01, ***P < 0.001. **B** CCK-8 assay was used to analyze the viability of MLE12 cells cultivated with different TMP concentrations prior to LPS treatment. Data from five individual experiments were analyzed using a one-way ANOVA and Bonferroni correction. Significant difference from TMP = 0 and LPS = 0 group: ^*^ *P* < *0.05*. Significant difference from TMP = 0 and LPS 10 ug/ml group: ^#^ *P* < *0.05*. **C**, **D** Levels of proinflammatory factors IL-1β and IL-6 in the cell supernatant in each group. **E**, **F** The levels of MDA and SOD activity indicated the oxidative stress status of each group. Data in (**C**)-(**F**) are expressed as mean ± SEM, and multiple comparisons were performed using one-way ANOVA with the Bonferroni coefficient (*n* = 6). *Significant difference compared with the control group, *P* < 0.05; ^#^Significant difference compared with the LPS group, *P* < 0.05; ^&^Significant difference from the LPS + TMP group, *P* < 0.05. ANOVA: analysis of variance; CCK-8: Cell Counting Kit-8; HO-1, heme oxygenase-1; KO, knockout; LPS, lipopolysaccharide; MDA: malondialdehyde; Nrf2: nuclear factor-erythroid 2-related factor 2; SOD: superoxide dismutase; TMP, tetramethylpyrazine

In endotoxin-induced ALI, alveolar epithelial cells have immunomodulatory and self-renewal abilities and play a vital role in lung repair [28]. Therefore, using MLE12 alveolar epithelial cells, we evaluated the effect of TMP on LPS-stimulated ALI in vitro. To simulate a cellular model of endotoxin-induced ALI, LPS was co-cultured with MLE12 cells at various doses over 24 h, and the CCK-8 assay was used to determine cell viability. As the concentration of LPS increased, cell viability gradually decreased (Fig. 6A). Cell viability decreased considerably when the LPS concentration was increased to 5 μg/ml. Therefore, 5 μg/ml was used as the LPS concentration in the experiments. MLE12 cells were treated with 5 μg/ml LPS for 24 h and pretreated with 2.5, 5, 10, 50, or 100 μg/ml TMP for 1 h. CCK-8 assays were then performed (Fig. 6B). A progressive increase in cellular viability was detected with 2.5, 5, 10, and 50 μg/ml TMP pretreatment. The highest effect was 83.74 ± 1.38% with 50 μg/ml TMP in LPS-treated cells (*P* < 0.05). Thus, 50 μg/ml TMP was selected for the subsequent experiments.

To explore the changes in the inflammatory level in each group, we determined IL-1β and IL-6 levels using the cell supernatant. Compared with control group, LPS group had considerably higher levels of IL-1β and IL-6, whereas TMP pretreatment reduced this trend (Fig. 6C, D). The levels of inflammatory factors were higher in Nrf2 siRNA + LPS + TMP group than in LPS + TMP group, indicating that the effect of TMP on LPS-induced inflammation was partially reversed by Nrf2 knockdown.

MDA levels and SOD activity were measured in each group to determine the degree of oxidative stress damage. Compared with LPS group, LPS + TMP group demonstrated lower levels of oxidative stress, as shown by lower MDA levels and higher SOD activity (Fig. 6E, F). However, Nrf2 knockdown partially attenuated the antioxidant stress effects of TMP in LPS-treated cells, with increased MDA levels and reduced SOD activity compared with the LPS + TMP group.

### TMP attenuated LPS-stimulated ALI by alleviating Golgi stress via the Nrf2/HO-1 pathway in vitro

**Fig. 7:**
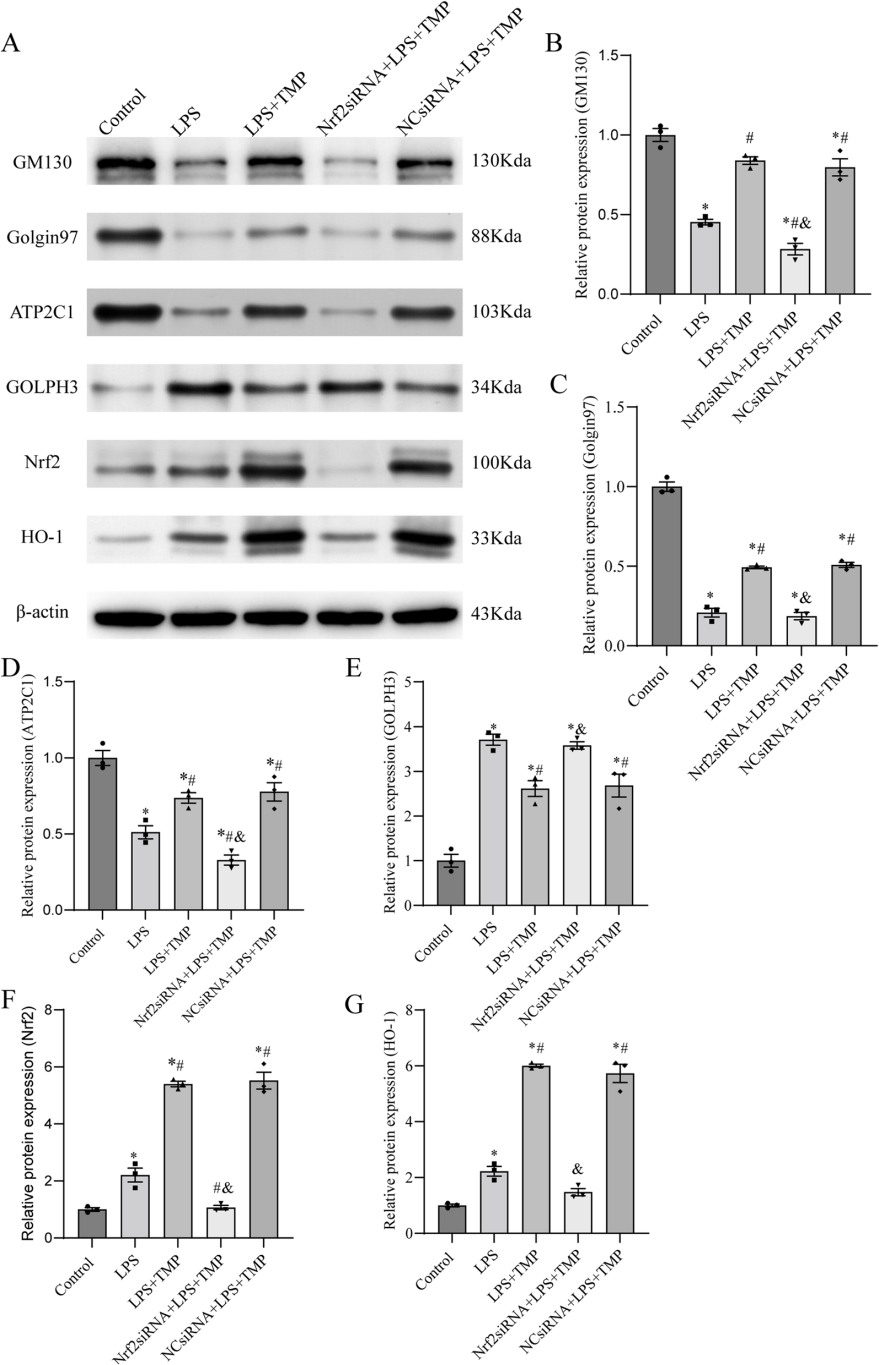
Nrf2 knockdown partially counteracted the Golgi stress-alleviating effect of TMP in LPS-stimulated MLE12 alveolar epithelial cells. **A**-**G** Representative western blotting and semi-quantification of Golgi stress-related (GM130, Golgin97, ATP2C1, and GOLPH3) and pathway-related (Nrf2 and HO-1) proteins. The blots were cropped for improving the clarity and conciseness of the presentation. Full-length blots were presented in Additional file 3. Band intensity analysis on western blotting images shows their relative ratio to β-actin. Data were expressed as mean ± SEM, and multiple comparisons were performed using one-way ANOVA with the Bonferroni coefficient (*n* = 3). *Significant difference compared with the control group, *P* < 0.05; ^#^Significant difference compared with the LPS group, *P* < 0.05; ^&^Significant difference from the LPS + TMP group, *P* < 0.05. ANOVA: analysis of variance; HO-1, heme oxygenase-1; LPS: lipopolysaccharide; Nrf2: nuclear factor-erythroid 2-related factor 2; SEM: standard error of the mean; TMP: tetramethylpyrazine

To further explore the mechanisms underlying the above findings, GM130, Golgin97, ATP2C1, and GOLPH3 expression and pathway-related protein Nrf2 and HO-1 expression were determined. Compared with LPS group, TMP pretreatment in LPS-stimulated cells significantly upregulated GM130, Golgin97, and ATP2C1 protein expression and downregulated GOLPH3 protein expression (Fig. 7A-E). Nrf2 siRNA + LPS + TMP group exhibited significantly higher levels of Golgi stress, as evidenced by decreased GM130, Golgin97, and ATP2C1 protein expression and increased GOLPH3 protein expression, compared with LPS + TMP group. TMP pretreatment upregulated Nrf2 and HO-1 protein expression, whereas Nrf2 knockdown reduced this effect (Fig. 7A, F-G). The above results demonstrated that TMP attenuated Golgi stress to alleviate endotoxin-induced ALI via the Nrf2/HO-1 pathway in vitro.

## Discussion

**Fig. 8:**
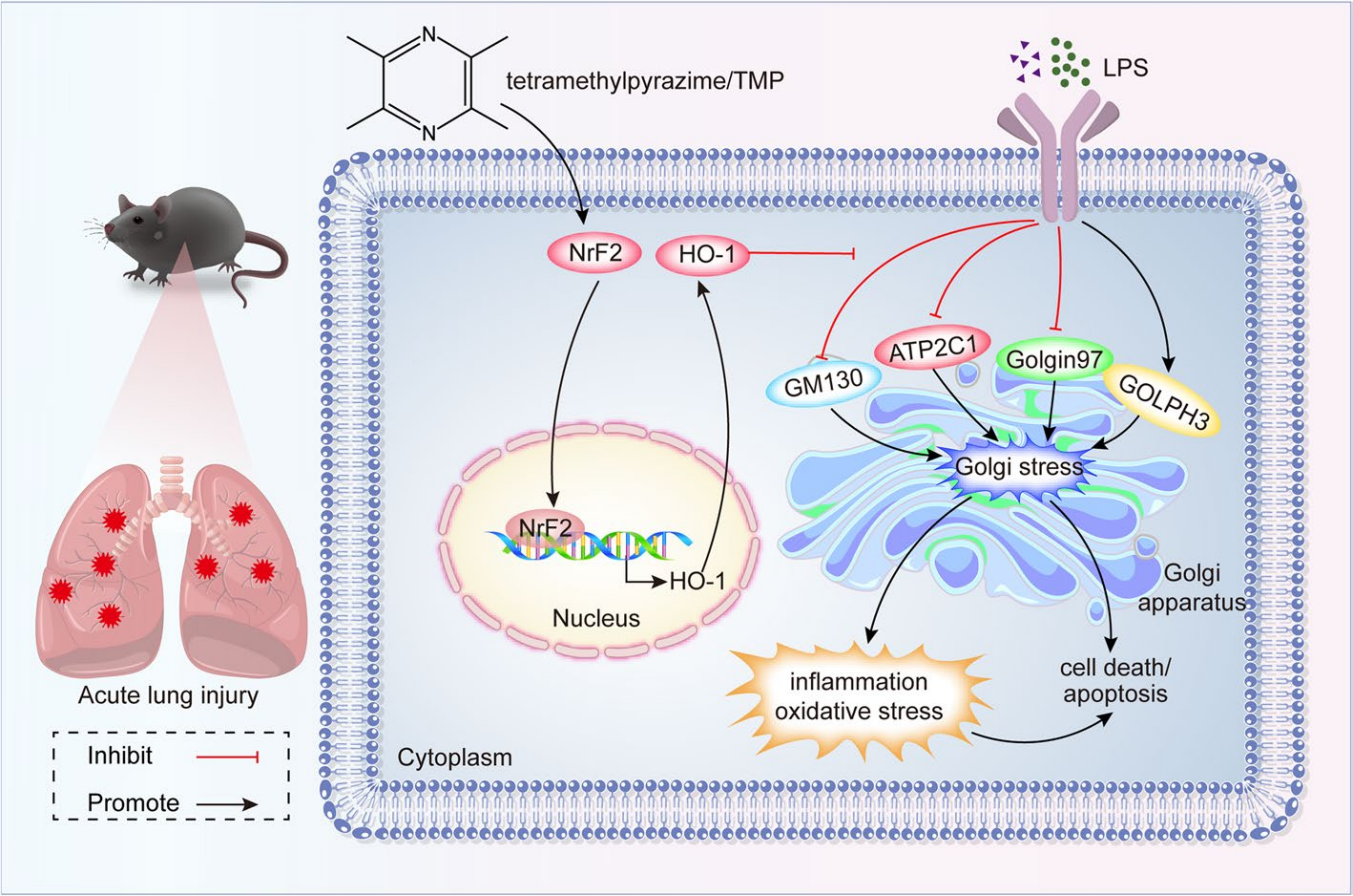
Graphical illustration of TMP pretreatment-mediated preservation of Golgi stress involved in regulating Nrf2/HO-1 signaling pathway. When LPS was administrated to C57BL/6J mice or MLE12 alveolar epithelial cells, TMP activated Nrf2/HO-1 pathway, leading to Nrf2 transferred from the cytoplasm to the nucleus, and increased the ARE response to induce target gene of HO-1 expression. Meanwhile, pretreatment of TMP upregulated the expressions of Golgi stress markers GM130, Golgin97, and ATP2C1, yet down-regulated the expression of Golgi stress-inducible protein GOLPH3. In conclusion, TMP pretreatment alleviates Golgi stress in endotoxin-related acute lung injury, thus alleviating the inflammation and oxidative stress of lung tissue, then reducing cell apoptosis or death

Our findings suggested that TMP alleviated endotoxin-induced ALI by reducing Golgi stress via the Nrf2/HO-1 signaling pathway. Previous research has shown that TMP can reduce ALI in sepsis via a variety of pathways, such as suppression of endoplasmic reticulum (ER) stress, apoptosis, and pyroptosis [23, 29]. Relevant research has not yet revealed the precise mechanism of the preventive impact of TMP on endotoxin-induced ALI. For the first time, we found that TMP attenuated LPS-stimulated pulmonary inflammation and oxidative stress damage via alleviating Golgi stress. In addition, the lung protective effect of TMP was partially carried out through Nrf2/HO-1 pathway (Fig. 8). Our findings may provide new approaches for the treatment of endotoxin-induced ALI.

### TMP alleviates LPS-induced ALI by inhibiting Golgi stress

ALI from sepsis can rapidly develop into increased pulmonary inflammation and damage to the alveolar-capillary barrier, even progressing to ARDS [30]. Previous studies have linked oxidative stress to the pathogenesis of sepsis and ALI [31–33]. Sepsis-induced ALI/ARDS, for which effective clinical treatment strategies are not available, is associated with a high mortality rate. Without exception, our research showed severe pathological damage, increased proinflammatory factors (TNF-1α, IL-1β, and IL-6), and greater oxidative stress (decreased GSH content and the GSH/GSSG ratio and increased GSSG content) in the LPS-stimulated ALI model. Therefore, identification of new treatments or mechanisms to treat endotoxin-induced ALI is urgently needed.

Golgi apparatus (GA), a complex and essential organelle in the cytoplasm, has received increasing attention based on its morphological and functional changes in several oxidative stress-related diseases [10, 12, 34]. GA response to oxidative stress has been labeled "Golgi stress" and is characterized as a stress-repair mechanism comparable to ER stress [10]. GM130 is a curved rod-shaped protein located in GA. It is required for several biological processes, including vesicle transport and mitosis [35, 36]. The trans-Golgi reticulum protein Golgin97 is required for Golgi stability and vesicle transport [13]. Both GM130 and Golgin97 participate in maintaining GA structure and are considered markers of Golgi stress. ATP2C1 is a Ca^2+^/Mn^2+^ ATPase specifically expressed in GA, and its depletion leads to an imbalance in intracellular Ca^2+^ homeostasis, increased oxidative stress, GA fragmentation, Golgi stress, and even cell death [10]. The reduction in ATP2C1 leads to an imbalance in Ca^2+^ homeostasis and fragmentation of GA. GOLPH3 is a peripheral membrane protein that is localized to the trans-GA [37]. Increased GOLPH3 expression has been noted in ischemia–reperfusion injury [38]. Elevated GOLPH3 expression further stimulates stress-related autophagy and ROS production. As a Golgi stress-inducible protein, GOLPH3 is positively associated with the degree of oxidative stress.

Based on previous studies, we selected GM130, Golgin97, ATP2C1, and GOLPH3 as markers of Golgi stress, and the changes in their expression levels indicated that Golgi stress was exacerbated or alleviated. Researchers have found that excessive Golgi stress may accelerate disease progressions, such as heterozygotes and skeletal muscle dysfunction [37, 39]. Notably, Golgi stress has been shown to have an important role in the pathological phase of endotoxin-induced ALI [13]. Interestingly, we found reduced GM130, Golgin97, and ATP2C1 expression and elevated GOLPH3 expression in the LPS-stimulated lung injury model. These findings implied that exacerbated Golgi stress is involved in LPS-stimulated lung injury. Therefore, drugs that target Golgi stress should be investigated to treat endotoxin-induced ALI.

TMP is a bioactive ingredient obtained from the traditional Chinese medicine chuanxiong, which was artificially synthesized in the 1970s [39]. TMP has been clinically applied in the treatment of stroke, heart attack, pulmonary hypertension, and other cardiovascular and cerebrovascular diseases owing to its vasodilation and inhibition of platelet aggregation effects [19, 40, 41]. Studies have demonstrated that TMP alleviates endotoxin-induced ALI in experimental models by exerting anti-inflammatory, antioxidative, and anti-apoptotic actions, but the mechanism has not been thoroughly elucidated [23, 41]. Our study found that TMP pretreatment attenuated LPS-stimulated ALI, as demonstrated by reduced lung pathology, decreased lung injury scores, lower levels of proinflammatory factors, and increased levels of oxidative stress. Furthermore, TMP pretreatment upregulated GM130, Golgin97, and ATP2C1 protein expression but downregulated GOLPH3 expression. Therefore, we concluded that TMP alleviated LPS-induced ALI by inhibiting Golgi stress.

### Nrf2/HO-1 pathway is implicated in TMP-mediated alleviation of Golgi stress and endotoxin-induced ALI

Nrf2 regulates intracellular protective antioxidants and redox reactions [42, 43]. Nrf2 activation is a critical mechanism for inhibiting ROS production and controlling oxidative stress. Furthermore, Nrf2 is considered an important moderator of ALI [44–46]. Activated Nrf2 enters the nucleus to bind to AREs and initiates the expression of downstream genes, including HO-1. HO-1 and its derivatives (carbon monoxide and biliverdin) have been proven to protect against inflammation and oxidative stress [47–49]. Moreover, researchers have found that HO-1 helps to protect the lungs by regulating Golgi stress [13]. According to our findings, TMP pretreatment enhanced Nrf2 and HO-1 expression and decreased LPS-induced lung damage. TMP exerted a pulmonary protective effect via the Nrf2/HO-1 pathway.

We used Nrf2 KO mice and Nrf2 siRNA-transfected MLE12 cells for further studies. Nrf2 knockout or knockdown partially reversed the protective effect of TMP pretreatment, based on increased pulmonary pathological injury, higher lung injury scores, and increased inflammatory and oxidative stress responses. Additionally, similar to GM130, Golgin97, and ATP2C1 expression, HO-1 protein expression was remarkably downregulated and GOLPH3 expression was upregulated in Nrf2 deletion models. These findings suggested that the Nrf2/HO-1 pathway is implicated in TMP-mediated alleviation of Golgi stress and endotoxin-induced ALI.

In conclusion, TMP pretreatment acted, at least partially, via the Nrf2/HO-1 pathway to inhibit Golgi stress to mitigate LPS-stimulated ALI in vivo and in vitro. TMP is clinically administered by intravenous infusion for 10–15 days as a course [39, 50], whereas a single intraperitoneal injection method of TMP was used in this experiment. Although the translational efficacy of TMP is debatable, this preclinical trial laid the framework for the use of TMP as a viable option for the prevention of sepsis-related lung injury.

## Conclusion

In conclusion, the key findings of our investigation were as follows: TMP attenuated LPS-stimulated pulmonary inflammation and oxidative stress damage by alleviating Golgi stress via the Nrf2/HO-1 pathway in vivo and in vitro. Selective suppression of Nrf2/HO-1 pathway reduced LPS-stimulated Golgi stress and inflammation. These data suggested that TMP is a viable therapeutic option for endotoxin-induced ALI.

### Supplementary Information

**Additional file 1.**

**Additional file 2.**

**Additional file 3.**

## Data Availability

The raw data supporting the conclusions of this article will be made available by the corresponding author without undue reservation.

## Abbreviations

TMP: Tetramethylpyrazine
LPS: Lipopolysaccharide
ALI: Acute lung injury
Nrf2: Nuclear factor erythroid 2-related factor-2
HO-1: Heme oxygenase-1
KO: Knockout
WT: Wild-type
GA: Golgi apparatus
GM130: Golgi matrix protein 130
ATP2C1: Golgi apparatus Ca^2+^/Mn^2+^ ATPases
GOLPH3: Golgi phosphoprotein 3
