# Supplementary figures and images for: Tetramethylpyrazine ameliorates endotoxin-induced acute lung injury by relieving Golgi stress via the Nrf2/HO-1 signaling pathway
Source: BMC Pulm Med. 2023 Aug 7;23:286. doi: 10.1186/s12890-023-02585-3 (PMC10408181; doi:10.1186/s12890-023-02585-3)

GM130 Figure 3

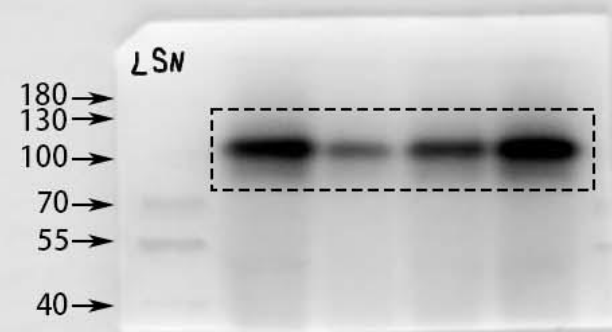

Golgin 97 Figure 3

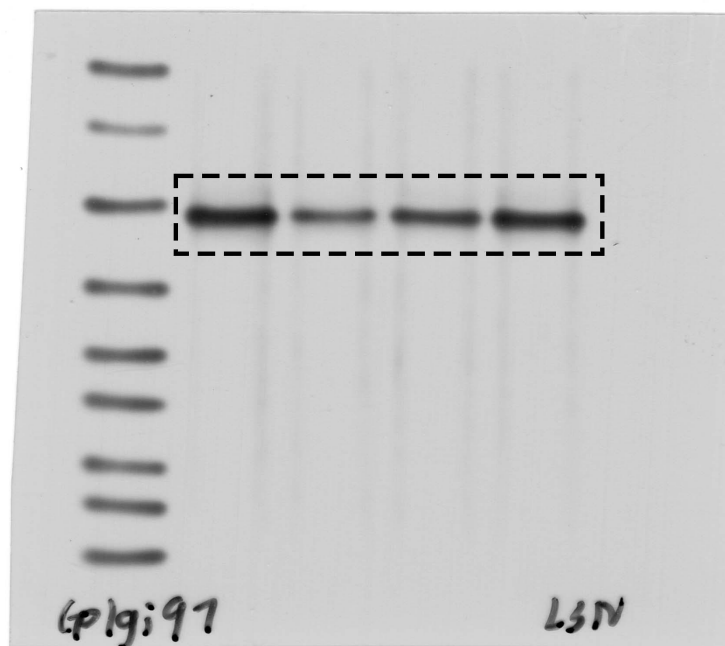

ATP2C1 Figure 3

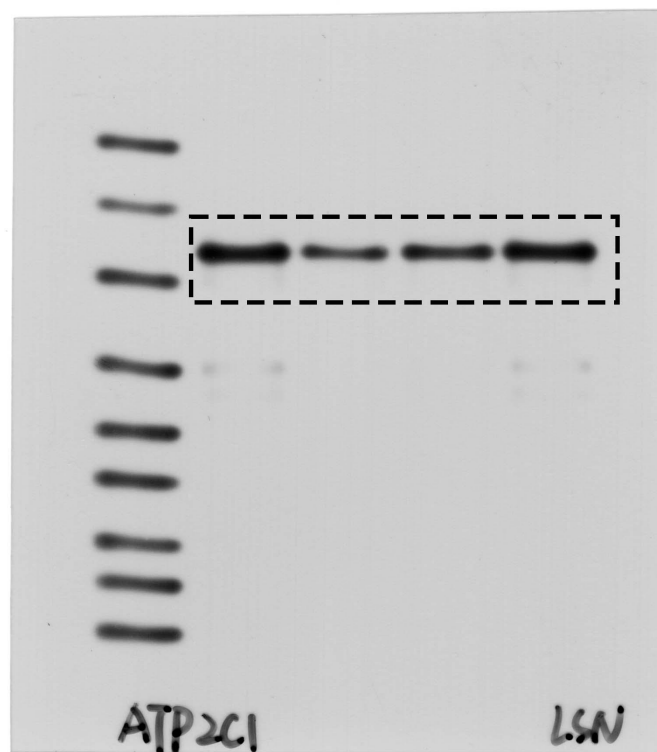

GOLPH3 Figure 3

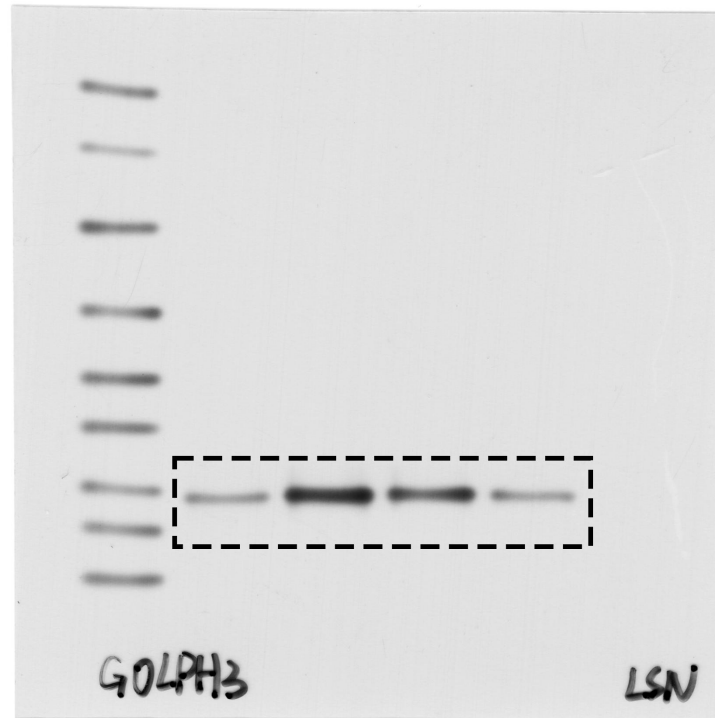

Nrf2 Figure 3

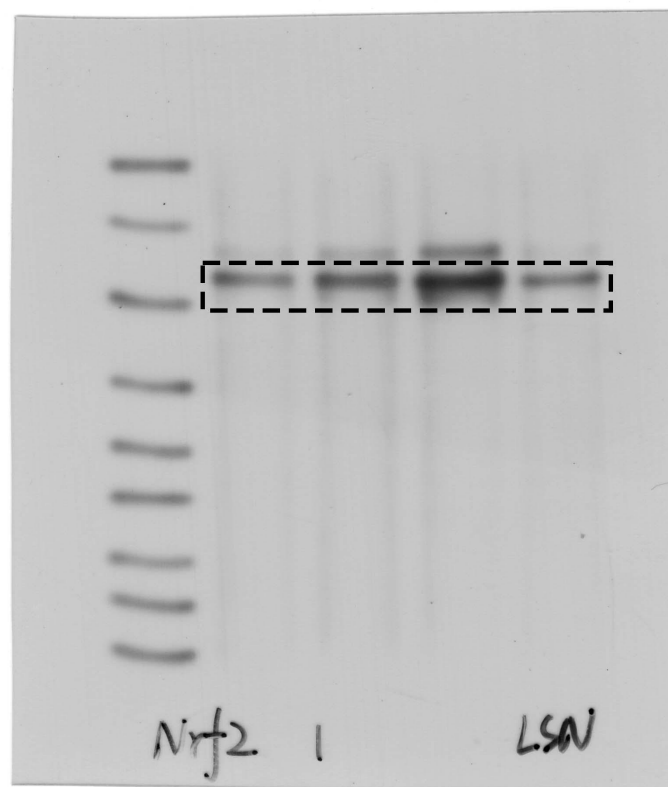

HO-1 Figure 3

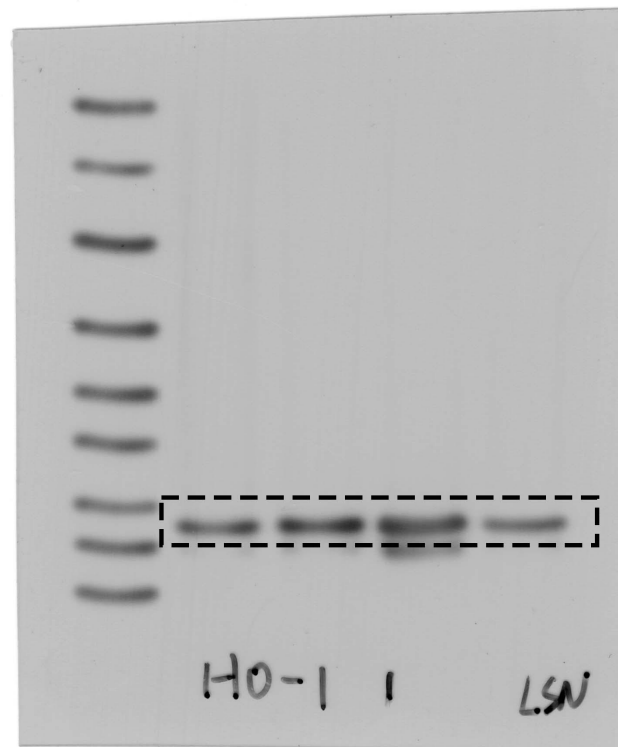

-actin Figure 3

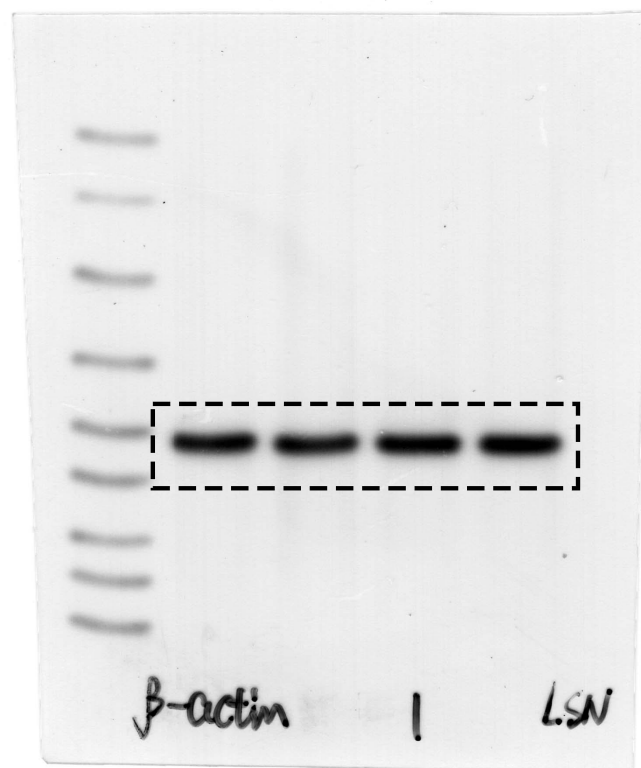

Supplement: Supplementary file 1 — Additional file 1. [file 12890_2023_2585_MOESM1_ESM.pdf]

GM130 Figure 5

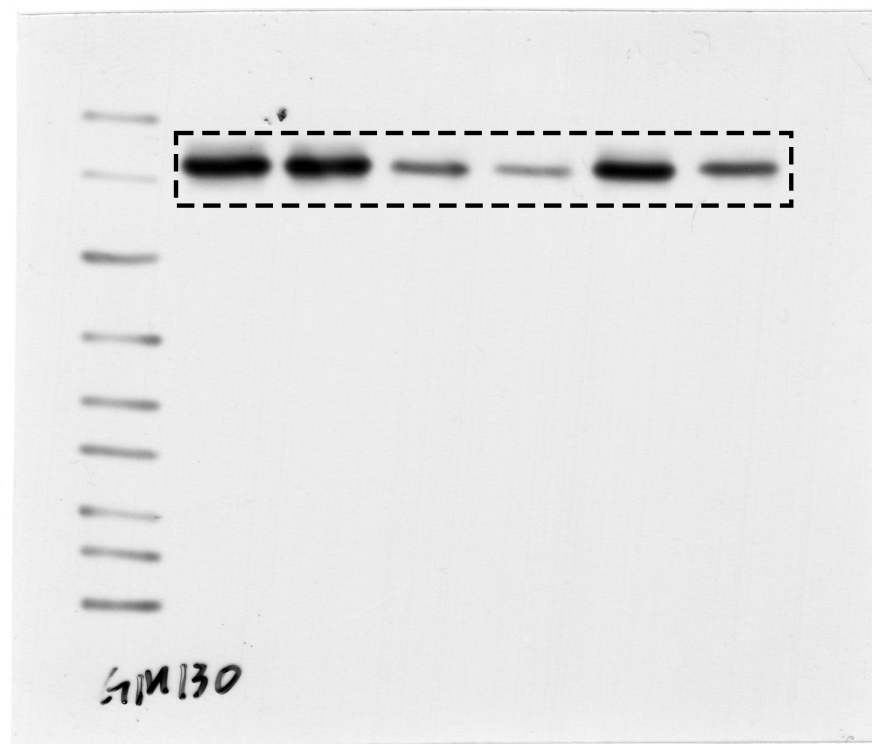

Golgin 97 Figure 5

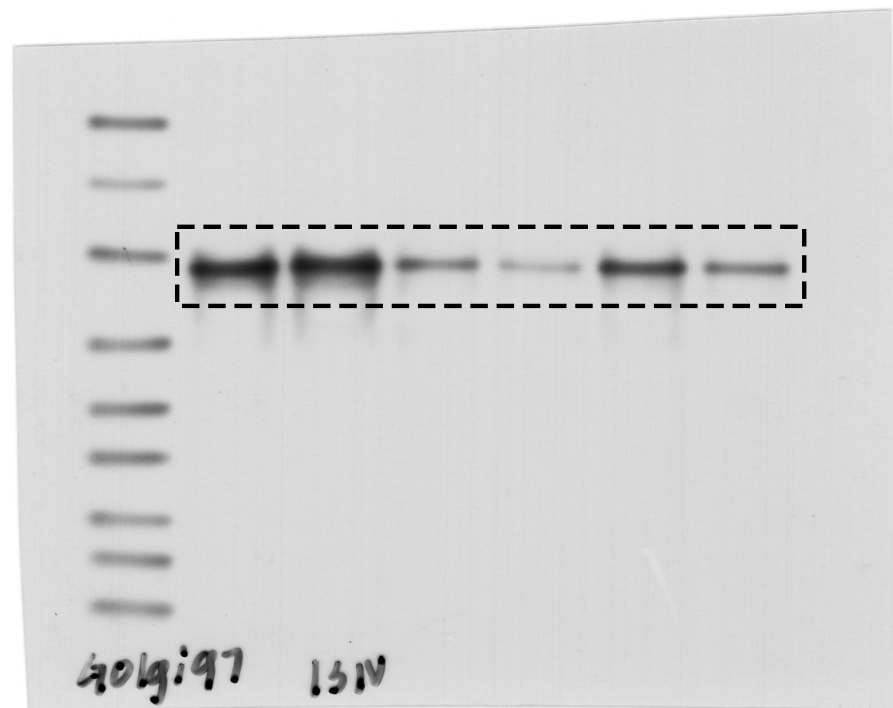

ATP2C1 Figure 5

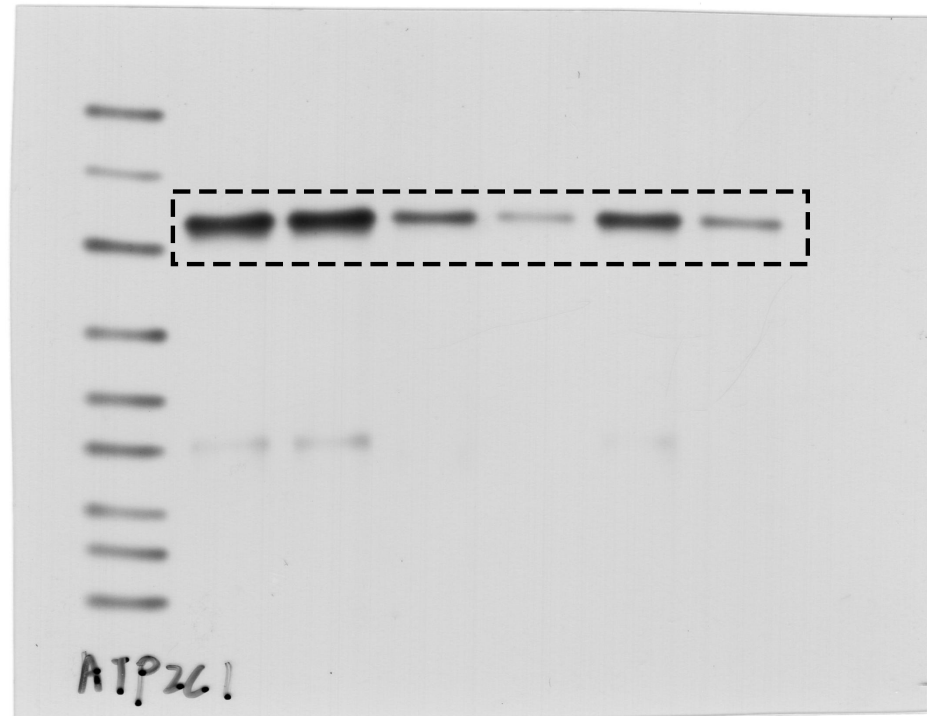

GOLPH3 Figure 5

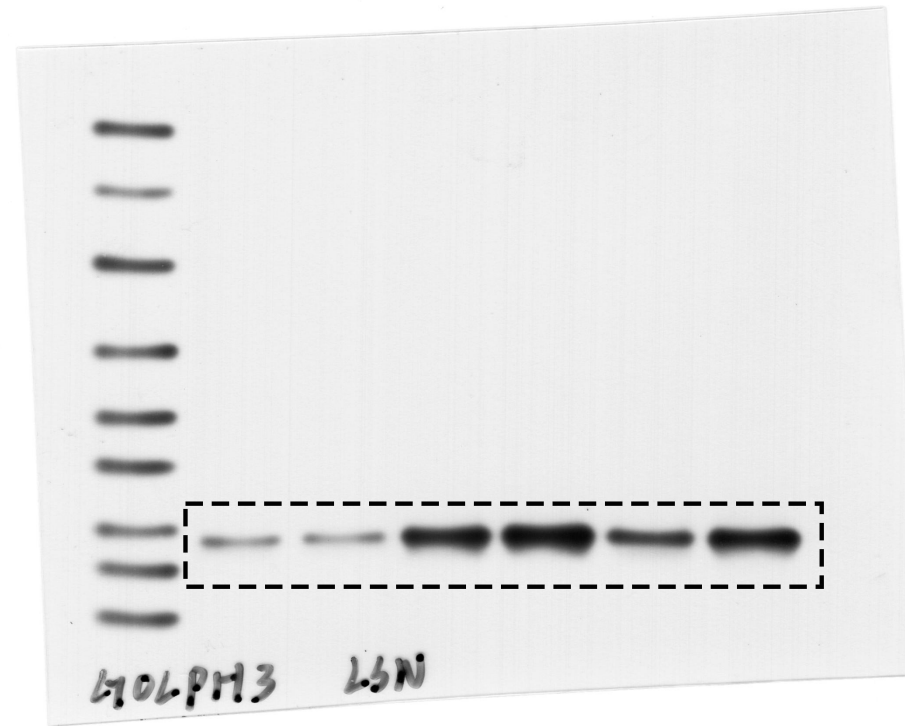

Nrf2 Figure 5

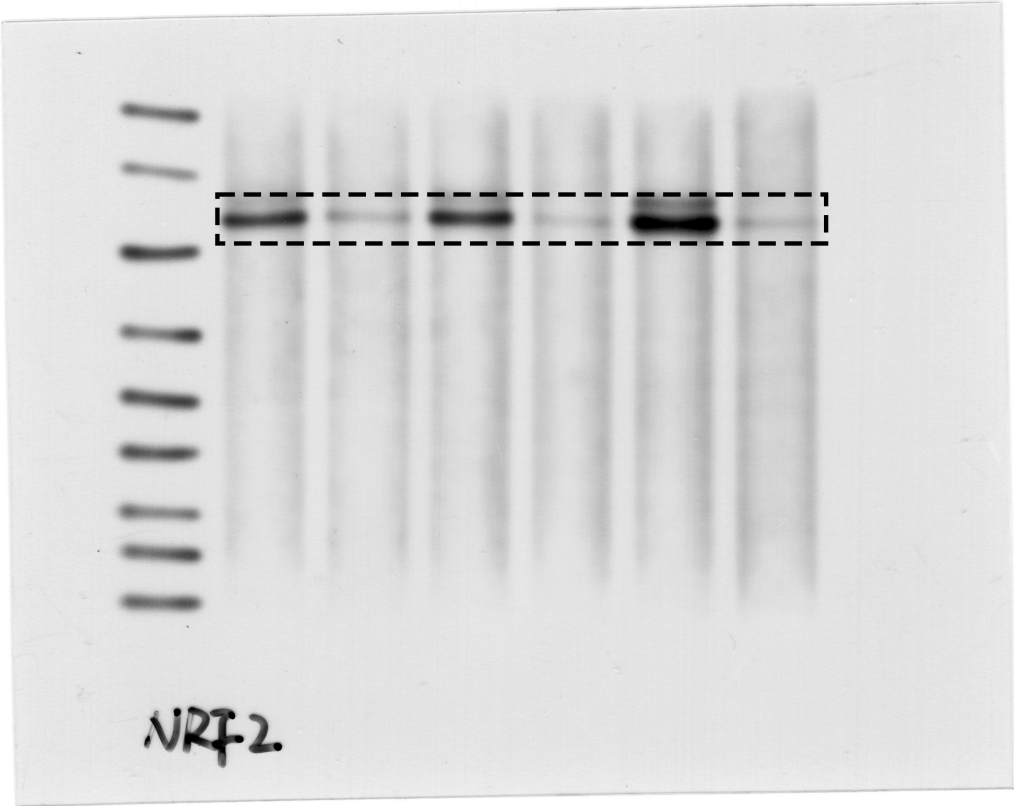

H0-1 Figure 5

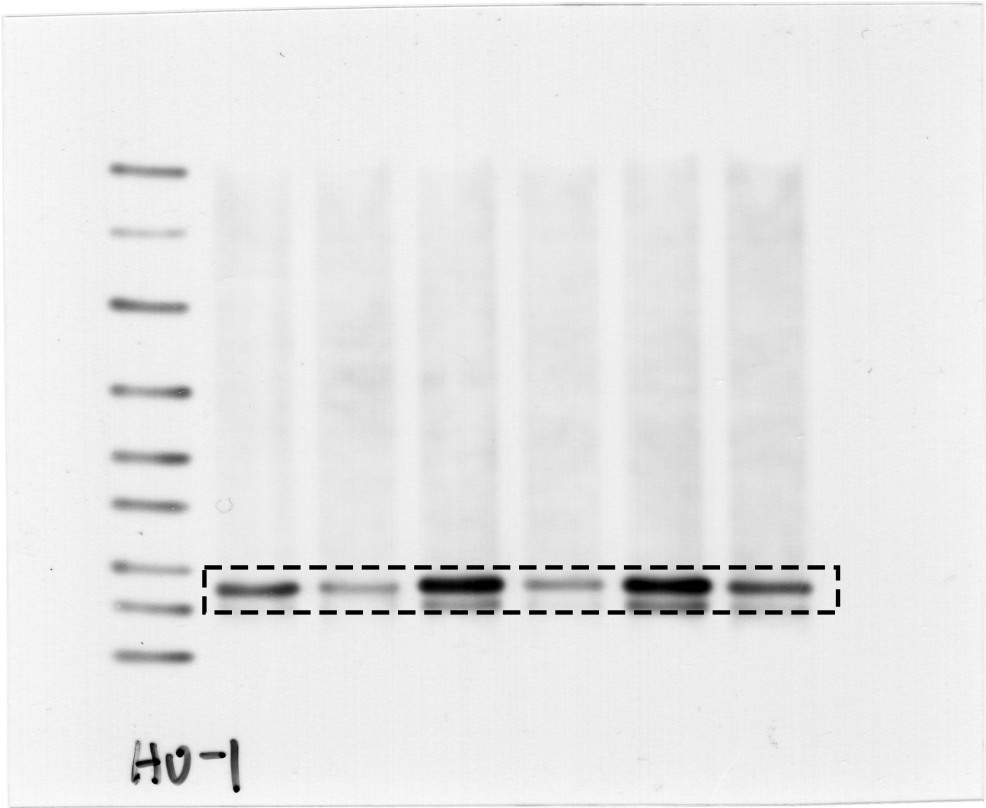

-actin Figure 5

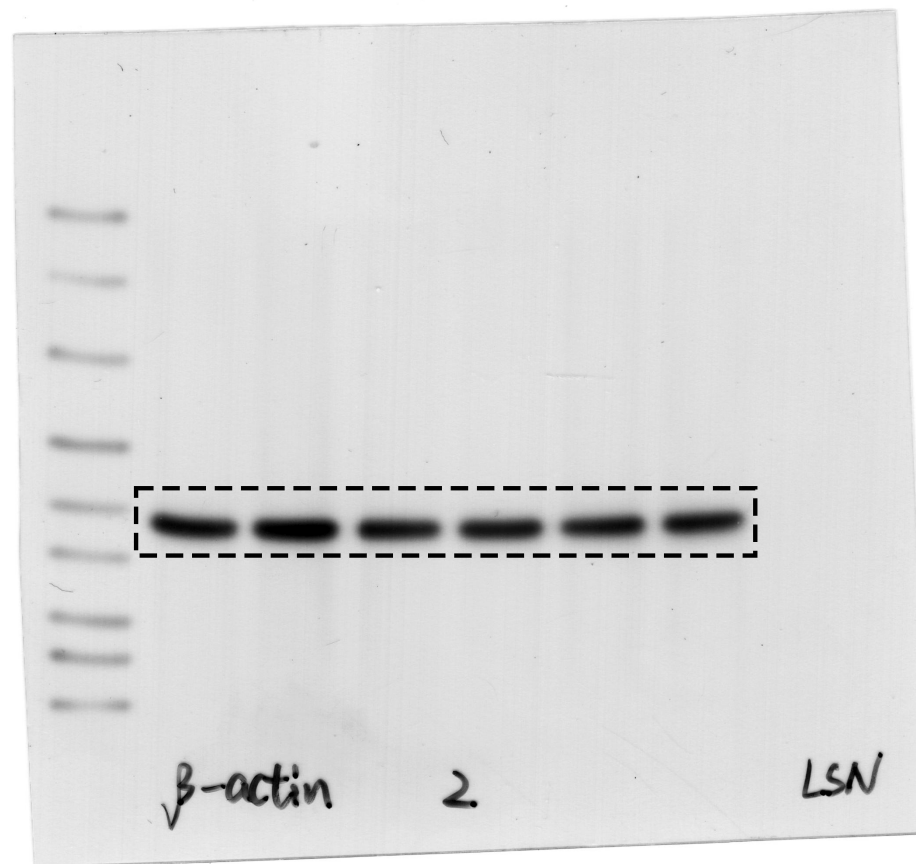

Supplement: Supplementary file 2 — Additional file 2. [file 12890_2023_2585_MOESM2_ESM.pdf]

GM130 Figure 7

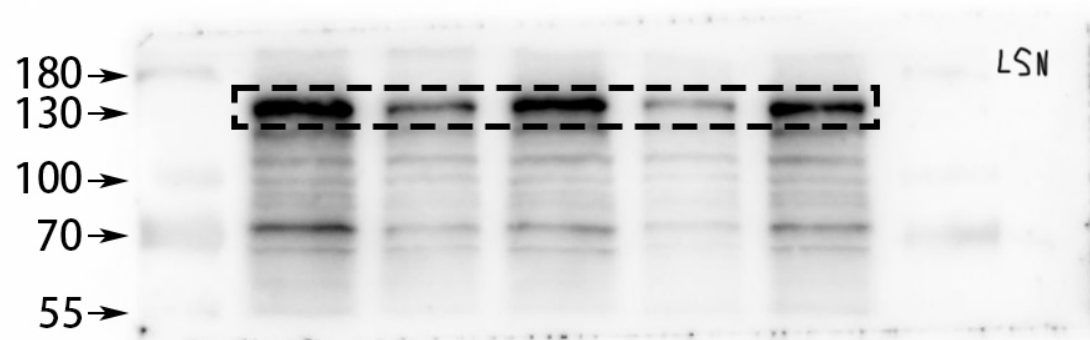

Golgin97 Figure 7

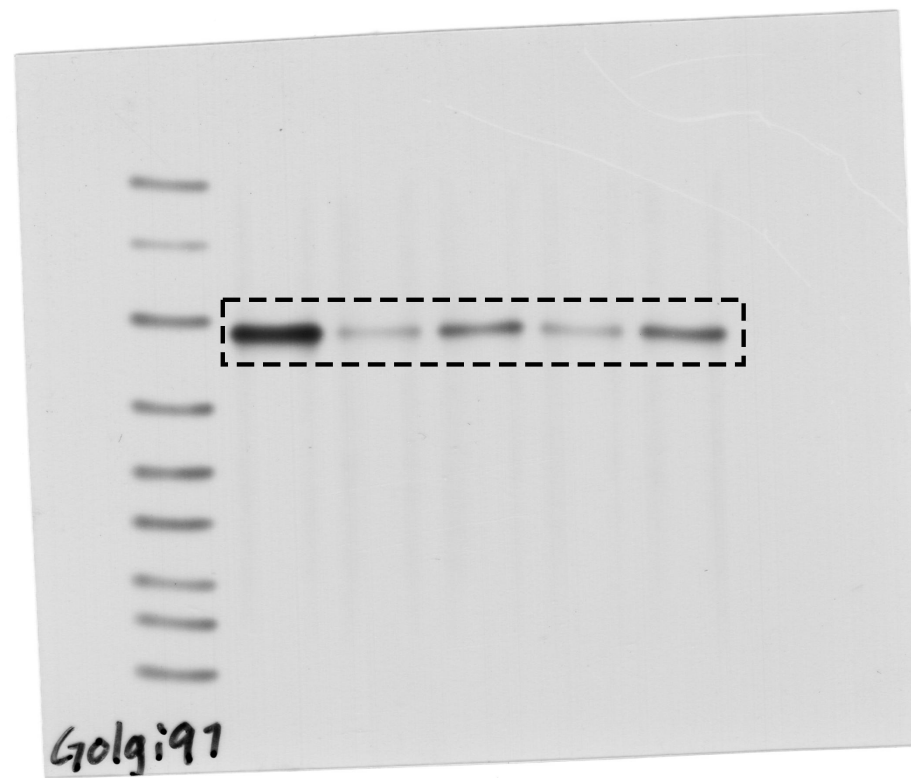

ATP2C1 Figure 7

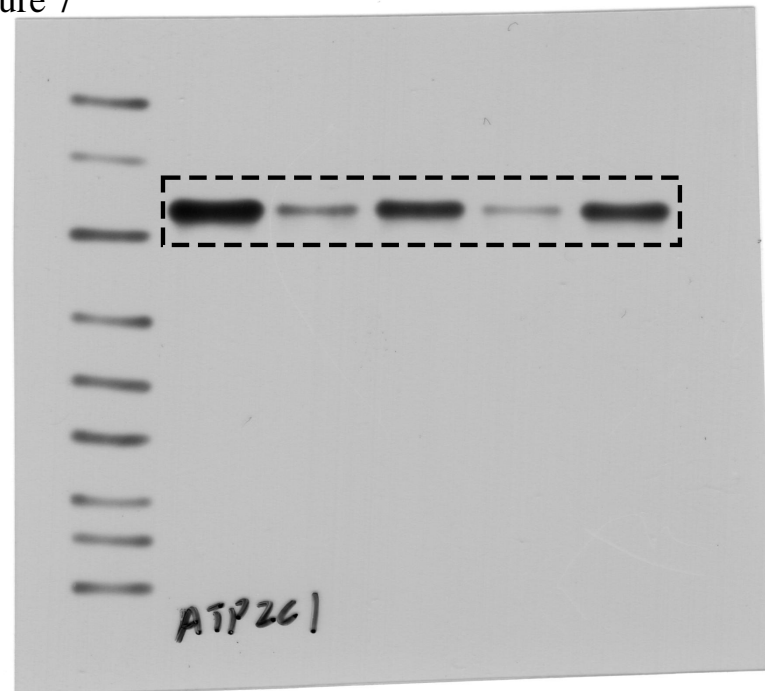

GOLPH3 Figure 7

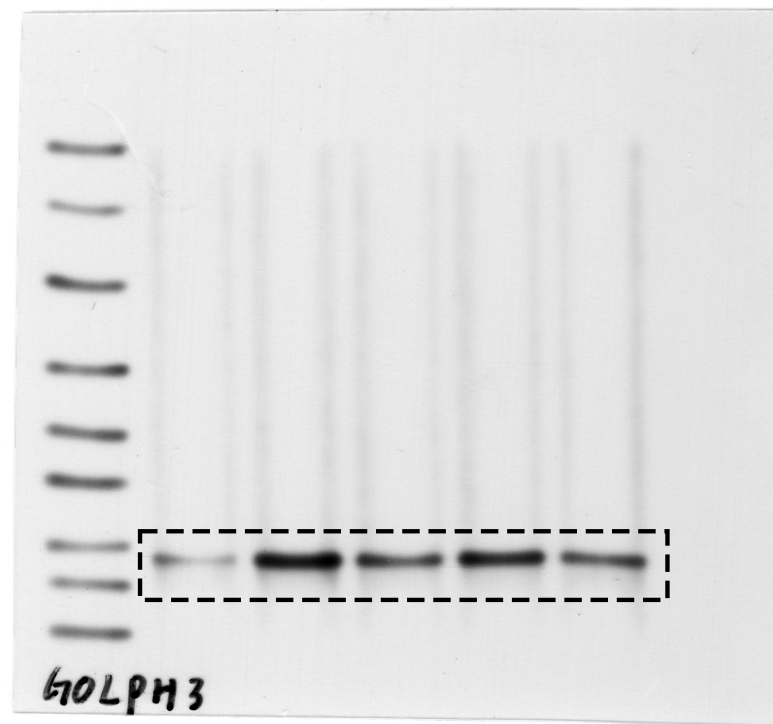

Nrf2 Figure 7

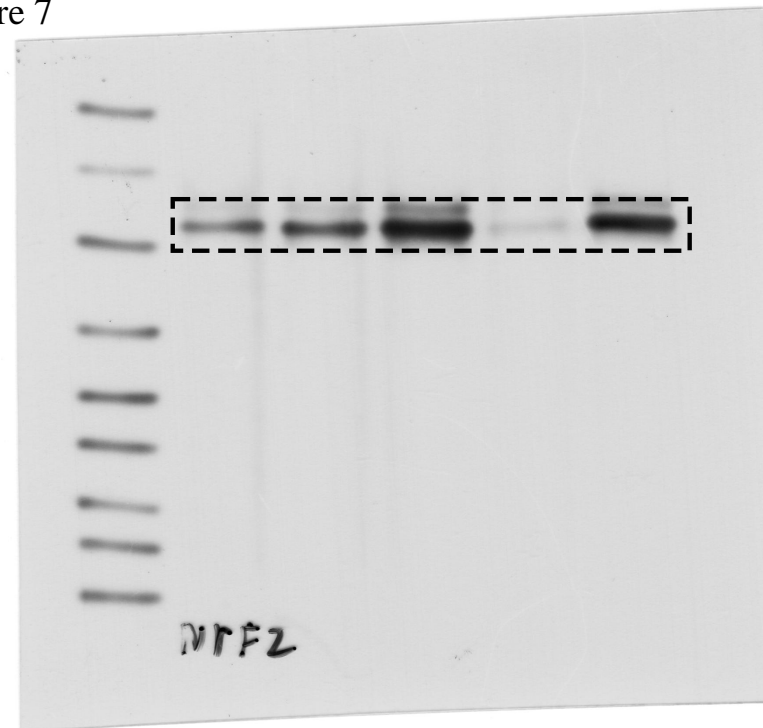

HO-1 Figure 7

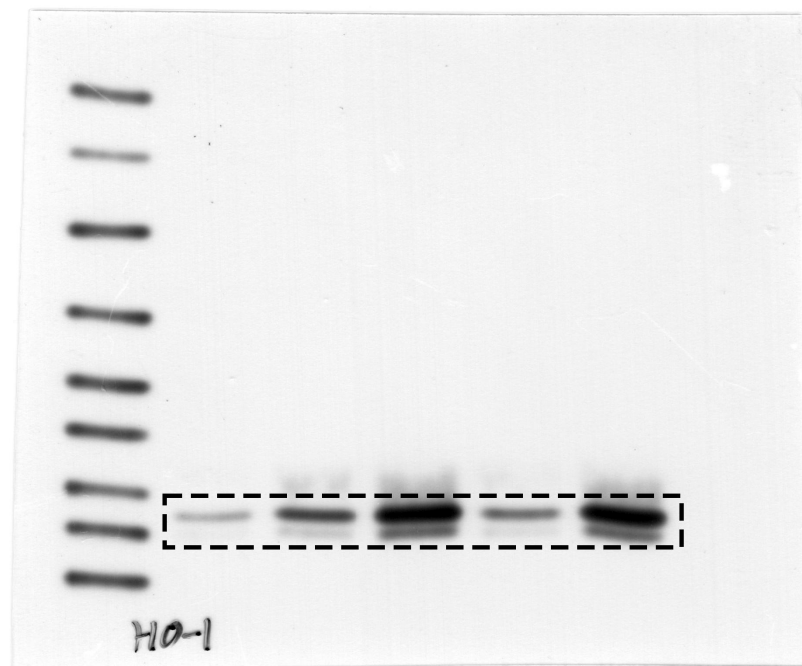

-actin Figure 7

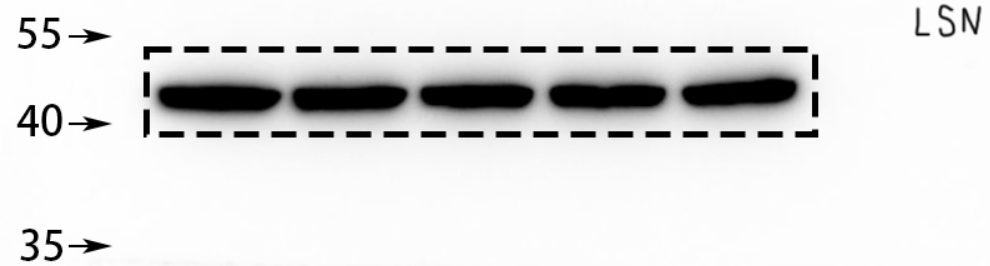

Supplement: Supplementary file 3 — Additional file 3. [file 12890_2023_2585_MOESM3_ESM.pdf]
